# Orientation Maps in V1 and Non-Euclidean Geometry

**DOI:** 10.1186/s13408-015-0024-7

**Published:** 2015-06-17

**Authors:** Alexandre Afgoustidis

**Affiliations:** Institut de Mathématiques de Jussieu-Paris Rive Gauche, Universite Paris 7 Denis Diderot, 75013 Paris, France

**Keywords:** Visual cortex, Orientation maps, Gaussian random fields, Euclidean invariance, Group representations, Non-Euclidean geometry, Pinwheel density, Kac–Rice formula

## Abstract

In the primary visual cortex, the processing of information uses the distribution of orientations in the visual input: neurons react to some orientations in the stimulus more than to others. In many species, orientation preference is mapped in a remarkable way on the cortical surface, and this organization of the neural population seems to be important for visual processing. Now, existing models for the geometry and development of orientation preference maps in higher mammals make a crucial use of symmetry considerations. In this paper, we consider probabilistic models for V1 maps from the point of view of group theory; we focus on Gaussian random fields with symmetry properties and review the probabilistic arguments that allow one to estimate pinwheel densities and predict the observed value of *π*. Then, in order to test the relevance of general symmetry arguments and to introduce methods which could be of use in modeling curved regions, we reconsider this model in the light of group representation theory, the canonical mathematics of symmetry. We show that through the Plancherel decomposition of the space of complex-valued maps on the Euclidean plane, each infinite-dimensional irreducible unitary representation of the special Euclidean group yields a unique V1-like map, and we use representation theory as a symmetry-based toolbox to build orientation maps adapted to the most famous non-Euclidean geometries, viz. spherical and hyperbolic geometry. We find that most of the dominant traits of V1 maps are preserved in these; we also study the link between symmetry and the statistics of singularities in orientation maps, and show what the striking quantitative characteristics observed in animals become in our curved models.

## Introduction

In the primary visual cortex, neurons are sensitive to selected features of the visual input: each cell analyzes the properties of a small window in the visual field, its response depends on the local orientations and spatial frequencies in the visual scene [1, 2], on velocities or time frequencies [3, 4], it is subject to ocular dominance [1], etc. These receptive profiles are distributed among the neurons of area V1, and in many species they are distributed in a remarkably orderly way [5–7]. For several of these characteristics (position, orientation), the layout of feature preferences is two-dimensional in nature: neurons form so-called *microcolumns* orthogonal to the cortical surface, in which the preferred simulus orientation or position does not change [1, 8]; across the cortical surface, however, the two-dimensional pattern of receptive profiles is richly organized [1, 5, 7–10].

**Fig. 1:**
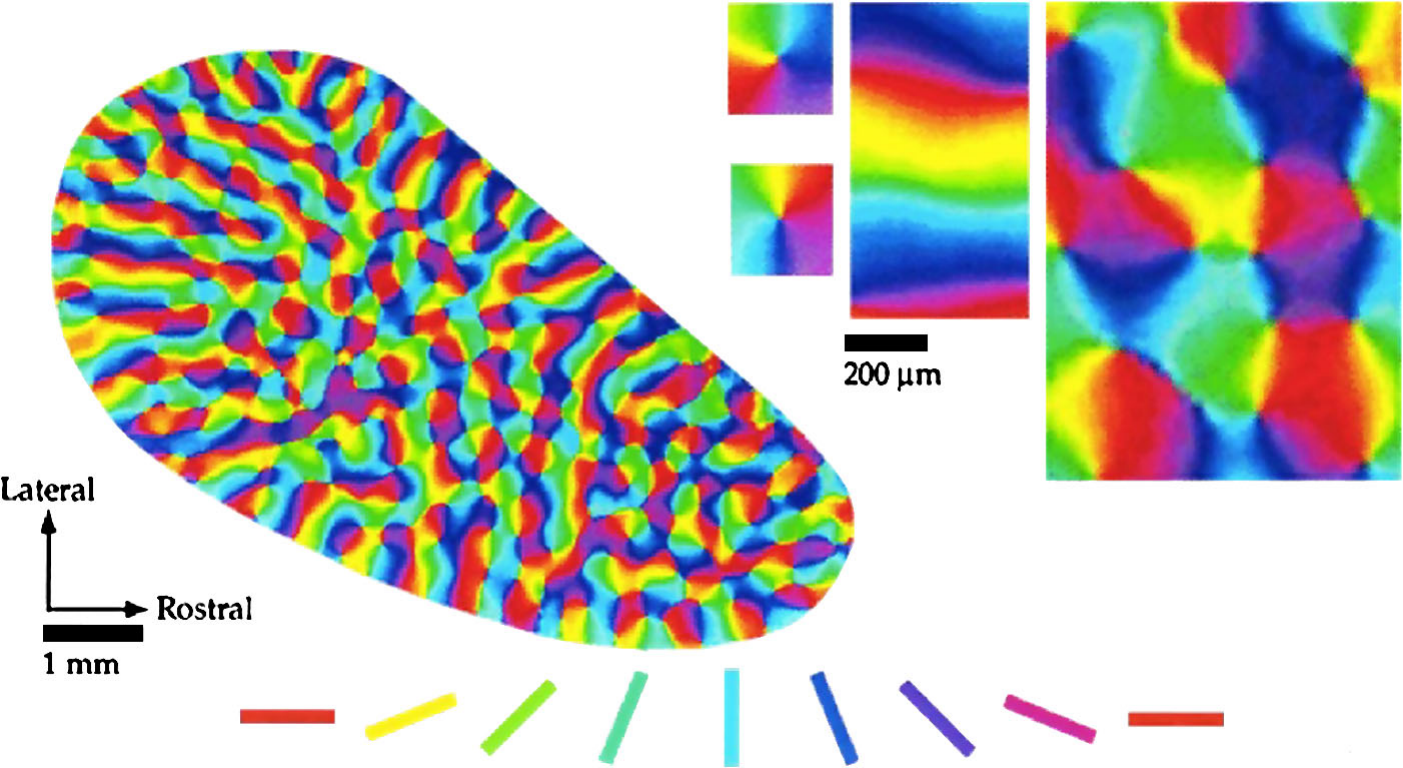
(Modified from Bosking et al. [7].) An Orientation Preference Map observed in the visual cortex of a tree shrew. The experimental procedure leading to this map is recalled in the main text. See also Swindale [18]. On the *upper right corner*, details at singular points (pinwheels) or regular points are shown

Amongst all feature maps in V1, it seems that the *orientation map* has a special part to play. Its beautiful geometrical properties (see Fig. 1) have prompted many experimental and theoretical studies (see [5, 7, 11–13]); the orientation map seems to be closely tied to the horizontal wiring (the layout of connectivities between microcolumns) of V1 [7, 14, 15], its geometry is correlated to that of all the other feature maps [16, 17], and while the geometrical properties of other feature maps vary much across species, those of orientation maps are remarkably similar [11].

It is thus tempting to attribute a high perceptual significance to the geometry of orientation maps, but is a long-standing mystery that V1 should develop this way: there are species in which no orientation map is present, most notably rodents [19, 20], though some of them, like squirrels, have fine vision [21]; on the other hand, it is a fact that orientation maps are to be found in distantly related species whose common ancestor likely did not exhibit regular maps. This has led to an intense (and ongoing) debate on the functional advantage of these ordered maps for perception, on the conditions under which such maps develop, and on the part self-organization has to play in the individual (ontogenetic) development of V1-like geometries [11, 20, 22, 23].

Our concern here is not with these general issues, but on geometrical principles that underlie some elements of the debate. We focus on models which have been quite successful in predicting precise quantitative properties of V1 maps from a restricted number of principles.

Our results will be based on methods set forth by Wolf, Geisel and others while discussing development models. They have shown that the properties of mature maps in a large region of V1 (that which is most easily accessible to optical imaging) are well reproduced by treating the mature map as a sample from a random variable with values in the set of possible orientation maps, and by imposing symmetry conditions on this random variable (see Sect. 2).

A remarkable chain of observations by Kaschube et al. [11, 24, 25] has shown that there are universal statistical regularities in V1 orientation maps, including an intriguing mean value of *π* for their density of topological defects (with respect to their typical length of quasiperiodicity; see Fig. 1 and Sect. 2). Wolf, Geisel and others [12, 26, 27] give a theoretical basis for understanding this; one of its salient features is the use of Euclidean symmetry.

In this discussion, the cortical surface is treated as a full Euclidean plane. Then conditions of homogeneity and isotropy of the cortical surface are enforced by asking for the probability distribution of the mentioned random variable to be invariant under translations and rotations of this plane. This is a condition of invariance under the action of the Euclidean group of rigid plane motions.

There are several reasons for wondering why the cortical surface should be treated as a Euclidean plane, and not as a curved surface like the ones supporting non-Euclidean geometries.

The underlying assumptions are not explicitly discussed in the literature. For instance, rigid motions can be considered in

- the geometry of the visual field,
- the geometry of the cortical surface, that of the actual biological tissue,
- or an intermediate functional geometry (e.g. treating motions of solid objects against a fixed background).

It is true that the part of V1 which is accessible to optical imaging is mostly flat, and that we may imagine an affine visual field to be flat as well.

However, we feel these three “planes” should be carefully distinguished, and that using Euclidean geometry simultaneously at all levels is not without significance.

A first, casual remark is that the way the (spherical) retina records the visual field uses its projective properties; it is on a rather functional level that we wan think of “the” affine visual field related to it by central projection from the retina (eye movements are amazingly well adapted to this reconstruction: see [28] for a discussion of motor computation in the Listing plane).

But more importantly, for almost all (if not all) animals which have been investigated, the correspondence between the accessible cortical region and the visual field (the retinotopic map) strongly departs from a central projection: it is logarithmic in nature, with a large magnification factor. For instance, even for the Tree Shrew which is known to have cortical V1 mostly flat, the observed region does not correspond to the center of the retina and the representation of the central field covers the major part of V1. A consequence is that Euclidean plane motions on the cortical surface and rigid motions in the visual field are very different. This is even more strikingly true for cats, primates and humans, whose calcarine sulcus has a more intricate 3D structure [29]. With this in mind, it seems very striking that the functional architecture of V1 should rely on a structure, the “association field” (see [13], Chap. 4) and its condition of “coaxial alignment” of orientation preferences, which simultaneously uses Euclidean geometry at several of these levels. It is also very interesting to note the successful use of shift-twist symmetry (see Sect. 3.2.4), a geometrical transformation which relates rotations on the cortical plane and rotations in the visual plane, in the study of hallucinatory patterns with contours [30] and of fine geometrical properties of V1 maps [31].

Thus when discussing plane motions, we feel that one should carefully keep track of the level (anatomical, functional, “external”) to which they refer. On the other hand, it is quite clear in Wolf and Geisel’s development models that the Euclidean invariance conditions are imposed at the cortical level, independently of the retinotopic map [11, 31].

Is then using flat *Euclidean* geometry at the cortical level indispensable? A closer look at the literature reveals that, when it appears, Euclidean geometry is endorsed only as a way to enforce conditions of *homogeneity and isotropy* on the two-dimensional surface of cortical V1. This makes it reasonable to look at the conditions of homogeneity and isotropy in non-Euclidean cases.

Now, these two notions are not at all incompatible with curvature; they are central in studying two-dimensional geometries with nonzero curvature, discovered and made famous by Gauss, Bolyai, Lobatchevski, Riemann and others. Extending the notions from geometry, analysis and probability to these spaces has been a source of great mathematical achievements in the late 19th and throughout the 20th century (Lie, Cartan, Weyl, Harish-Chandra, Yaglom). The central concept is that of transformation group, and the corresponding mathematical tools are those of noncommutative harmonic analysis, grounded on Lie group representations. In fact, Wolf and Geisel’s ingredients precisely match the basic objects of invariant harmonic analysis.

Our aim in this paper is to use these tools to define natural V1-like patterns on non-Euclidean spaces. Because symmetry considerations are central to the whole discussion, we need our non-Euclidean spaces to admit enough symmetries for the conditions of homogeneity and isotropy to make sense, and we thus consider the two-dimensional symmetric spaces. Aside from the Euclidean plane there are but two continuous families of models for such spaces, isomorphic to the sphere and the hyperbolic plane, so these two spaces will be the non-Euclidean settings for our constructions.

The success of Euclidean-symmetry-based arguments for describing flat parts of V1 makes it quite natural, from a neural point of view, to wonder whether in curved regions of V1, the layout of orientation preferences develops according to the same principles, and what could be the importance of the metric induced by cortical folding or of “coordinates” which would be induced by flattening the surface (and with respect to which the notion of curvature loses its meaning). It is a matter of current debate whether the three-dimensional structure induced by cortical folding has functional benefits; present understanding seems to be that its structure is the result of anatomical constraints (like the tension along cortico-cortical connections, or the repartition of blood flow; see [32]), but several hypotheses have been put forward to assess its functional meaning (see for instance [32, 33]). In a study trying to assess the importance of cortical folding for orientation maps it would be natural of course to consider variable curvature, but it is difficult to see how symmetry arguments could generalize and even make sense, whereas in regions having large (local) symmetry groups we shall see that it is very natural to adapt the successful arguments for flat V1 after a suitable interpretation of the latter. As we shall point out in the upcoming Discussion, there might also be benefits (in terms of information processing) in having symmetry groups as large as possible in rather extended regions.

Here is an outline of the paper. In Sect. 2, we first proceed to describe some aspects of Wolf and Geisel’s models with the words of representation theory; in this situation the relevant group is the Euclidean group of rigid plane motions. We introduce the probabilistic setting to be used in this paper, that of Gaussian random fields, in Sect. 2.1, and discuss the crucial Euclidean symmetry arguments in Sect. 2.2. We bring group theory into the picture in Sect. 2.3, and irreducible representations in Sect. 2.4.

To pass over to non-Euclidean geometries, we then examine what happens if the Euclidean group is replaced by the isometry groups of other symmetric spaces; we thus define “orientation maps” on surfaces of negative or positive curvature. For symmetric spaces the curvature is a numerical constant, and after a renormalization the two-dimensional symmetric spaces turn out to be isomorphic with the Euclidean plane, the hyperbolic plane or the round sphere. We begin Sect. 3 with the hyperbolic, negatively curved setting rather than the spherical, positively-curved one, because there are closer links with flat harmonic analysis in that case. After introducing our orientation-preference-like maps on these spaces, we emphasize the important part symmetry plays in the existence of the universal value for defect (pinwheel) densities in V1 maps by discussing the density of topological defects in non-Euclidean orientation maps.

As we shall see, in the Euclidean case, irreducible representations enter the picture through the existence of a dominant wavelength in the correlation spectrum; our recent paper in this journal [34] focuses on the role of this monochromaticity condition in getting a precise pinwheel density and quasiperiodicity. Although some of our results can find motivation from a few remarks in that paper, the present study is independent from [34].

## Methods

### Gaussian Random Fields

How was the map of Fig. 1 obtained [7]? High-contrast square wave gratings were presented to the animal, and optical imaging was used to measure the difference between the responses of neurons on the cortical surface upon translation of the visual input. From these data, a pattern emerges that attributes, given a stimulus orientation *ϑ*, a sensitivity $a_{\vartheta}(x)$ to every point *x* of the cortical surface (so $a_{\vartheta}$ is a positive-valued continuous function on the cortical surface $\mathcal {X}$). If this is recorded for a number of directions $\vartheta_{1}, \ldots, \vartheta_{N}$, and if the column beneath a point $x_{0} \in \mathcal{X}$ of the cortical surface has orientation preference $\vartheta_{j}$, then the polygon whose vertices are the points $a_{\vartheta_{k}}(x_{0}) e^{2i\vartheta_{k}}$ in $\mathbb {C}$ will be elongated in the direction $2\vartheta_{j}$, and the argument of the complex sum

$$\mathbf{z}_{\mathrm{exp}}(x_{0}) = \sum_{k=1}^{N} a_{\vartheta_{k}}(x_{0}) e^{2i\vartheta_{k}} $$

will be approximately $2\vartheta_{j}$. The functions $a_{\vartheta_{k}}$ for a tree shrew V1 were obtained using optical imaging, and the map drawn on Fig. 1 is simply $x \mapsto\frac{1}{2} \arg\mathbf{z}_{\mathrm{exp}}(x)$.

Let us add that if there is a pinwheel center at $x_{0}$, by definition^1^$\mathbf{z}_{\mathrm{exp}}$ takes all values of the argument in a neighbourhood of $x_{0}$, so $\mathbf{z}_{\mathrm{exp}}(x_{0})$ must be zero. On the other hand, the modulus of $\mathbf {z}_{\exp}$ may loosely be interpreted as a measure of orientation selectivity: when orientation tuning at $x_{0}$ is poor, all of the $a_{\vartheta_{k}}(x_{0})$ will be approximately the same, so $x_{0}$ will be close to a zero of $\mathbf{z}_{\mathrm{exp}}$, while if orientation selectivity at $x_{0}$ is sharp, the numbers $a_{\vartheta_{k}}(x_{0})$ for which $\vartheta_{k}$ is close to the preferred orientation will be much larger than the others, and the modulus of **z** will be rather high at $x_{0}$.

With this interpretation, we may discuss any complex-valued smooth function **z** on a surface *X* as if its argument were an orientation map, and its modulus were a measure of orientation selectivity. Orientation selectivity near pinwheel centers is being actively researched and debated, see [9, 35] and the references in [36], so interpreting the modulus of the vector sum $z_{\mathrm{exp}}$ in this way might be questioned, but this tradition dates back to 1982 [18].

If mathematical models yielding plausible maps *z* are to be furnished, then certainly they should be compared to the multitude of maps observed in different individuals. Let us neglect, for a given species, the slight differences in cortical shape and assume that each test subject comes with a coordinate system on the surface of its V1, so that we may compare a given map from $\mathbb {R}^{2}$ to $\mathbb {C}$ to the orientation map observed in this individual.

We can then compare the different individual maps, leading to *map statistics*; if orientation maps are to be described mathematically, it seems fair to hope for a mathematical object that produces, rather than a single complex-valued function with the desired features, statistical ensembles of realistic-looking maps [12]. This approach might not be the best way to account for the finer properties of mature maps as experimentally observed, and it is certainly a rough approximation that needs to be confronted with the output of more biologically plausible development models. However, it does have the advantage of mathematical simplicity, and as we shall see, it is particularly well suited to discussing the part symmetry arguments have to play in producing realistic maps.

So what we need is a *random field*, that is, a random variable with values in the set of smooth maps from $\mathbb {R}^{2}$ to $\mathbb {C}$. Since the set of smooth maps is infinite-dimensional, we cannot expect to find interesting “probability distributions” from closed formulae [37, 38]; but in the case of V1, the general theory of random fields and the available biological information make it possible to describe special fields whose “typical realizations” yield rather realistic maps [26, 38]. When we go over to non-Euclidean settings in this paper, we shall see that the mathematical description can be adapted to provide special random fields defined on non-Euclidean spaces; their typical realizations will yield V1-like maps adapted to the considered non-Euclidean geometry.

But let us now make our way toward the special fields on Euclidean space whose typical realizations look like orientation maps.

Measured statistical properties of real orientation maps include correlation functions [31]: it turns out that the structures of correlation measured in different individuals look very much alike. This is important: many discussions take the architecture of correlations to be essential to the horizontal wiring of V1, and to be at the heart of its perceptual function [13, 39]; it is also at the heart of striking results on the distribution of singularities in OPMs [11, 12, 26]. So using models that reproduce this correlation structure seems to be a good idea, and there is a way to associate special random fields to correlation structures.

#### Definition

A complex-valued random function **z** on a smooth manifold *M* (a collection $\mathbf{z}(x)$, $x \in M$ of complex-valued random variables) is a *complex-valued centered Gaussian random field* (GRF) if, for every integer *n* and every *n*-tuple $(x_{1}, \ldots ,x_{n}) \in M^{n}$, the $\mathbb {C}^{n}$-valued random variable $(\mathbf{z}(x_{1}), \ldots, \mathbf{z}(x_{n}))$ is Gaussian with zero mean. Its *correlation function*$C: M^{2} \rightarrow \mathbb {C}$ is the (deterministic) map $(x, y) \mapsto\mathbb{E} [ \mathbf {z}(x) \bar{\mathbf{z}}(y) ]$.

Just as a Gaussian probability distribution on $\mathbb {R}$ is available when a value for expectation and a value for variance are given (and is the “best bet”, that is, the minimum entropy distribution, given these data [40]), a continuous two-point correlation function $C: M^{2} \mapsto \mathbb {C}$ (together with the zero-mean requirement in the definition we use here) determines a unique GRF thanks to an existence theorem by Kolmogorov: see [41], Theorem 12.1.3, [38], and [37], p. 4.^2^

In what follows, we shall always require that $C: M^{2} \rightarrow \mathbb {C}$ be smooth enough; in fact we will only meet fields with real-analytic correlation functions. Maps drawn from such fields are almost surely smooth, so there is no regularity problem ahead.

Before we add symmetry constraints on our Gaussian fields, note that $C(x, x)$ is the variance of $\mathbf{z}(x)$; this depends on a choice of unit for measuring orientation selectivity. We shall proceed to a convenient one in the next subsection.

### Euclidean Symmetry in V1

Let us for the moment deal with the cortical surface as if it were an Euclidean plane $\mathbb {R}^{2}$. In a grown individual, different points on this plane correspond to neurons that usually do not have the same orientation preference, whose connectivity reaches out to different subsets of the cortex [13, 30, 39]; at some points we find sharp orientation tuning and under others (pinwheels) a less clear behaviour. In short, two different points on the cortical surface usually have different parts to play in the processing of visual information. But experimental evidence [7, 42] suggests very clearly that no particular point on this plane should have any distinguished part to play in the *general design* of the orientation map (e.g. be an organizational center for the development of the map, or have a systematic tendency to exhibit a particular orientation preference in the end).

These two facts are *not* incompatible: we may use this homogeneity condition as a constraint on the *ensemble properties* of the Gaussian field we are trying to obtain realistic maps from. In other words, given a possible outcome $x \mapsto z(x)$ for **z**, we may require that

$$x \mapsto z(x+u) \quad \bigl(\text{where }u\text{ is any vector in } \mathbb {R}^{2} \bigr) $$

and

$$x \mapsto z(Rx) \quad(\text{where }R\text{ is any } 2 \times2 \text{ rotation matrix}) $$

have the same occurrence probability as $x \mapsto z(x)$. Rotations and translations come together in the *Euclidean group*$\mathit{SE}(2)$, which is the set of transformations of the plane that preserve Euclidean distance and the orientedness of bases [43–45]; an element *g* of this group is easily shown to be uniquely specified by a couple $(R,u)$ where *R* is a rotation matrix and *u* a vector, and it is readily checked that elements $g_{1}=(R_{1},u_{1})$ and $g_{2}=(R_{2},u_{2})$ compose as $g_{1} g_{2}= ( R_{1}R_{2}, u_{1} + R_{1}u_{2} )$.

The above assumption is then that *the probability distribution of***z***is invariant under the action of E(2)* on the set of maps [26].

This implies that $C(x,y)$ depends only on $\|x-y\|$, and in the case of a Gaussian field this apparently weaker form of invariance is actually equivalent to the invariance of the full probability distribution. Let us write $\varGamma: {\mathbb {R}^{2}} \rightarrow \mathbb {C}$ for the radial function such that $C(x,y) = \varGamma(x-y)$ for all *x* and *y*, and note that up to a global rescaling of the modulus used to measure orientation selectivity, we may (and will) assume $\varGamma(0) = 1$.

Further discussion of correlations may be conducted using *Γ*, and there is an important remark to be made here: the high-frequency components of its Fourier transform record *local* correlations, while low-frequency components in $\widehat{\varGamma}$ (the Fourier transform of *Γ*) point to long-range correlations. If **z** is to produce a quasi-periodic layout of orientation preferences with characteristic distance *Λ*, this seems to leave no room for systematic correlations at a much longer or much shorter distance than *Λ*. So it seems reasonable to expect that Gaussian fields generating plausible maps have $\widehat{\varGamma}$ supported on the neighbourhood of a circle with radius $\frac{2\pi}{\varLambda}$. Following Niebur and Worgotter, Wolf and Geisel and others, we note that *this further hypothesis on*$\widehat{\varGamma}$*is all that is needed to generate realistic-looking maps*.

The simplest way to test this claim is to use what we shall call a *monochromatic invariant random field*, a field in which $\widehat {\varGamma}$ actually has support in a single circle, and consequently is the Dirac distribution on this circle:^3^$\varGamma(\vec{r}) = \int_{\mathbb {S}^{1}} e^{i \frac{2\pi}{\varLambda} \vec{u} \cdot\vec{r}} \,d\vec{u}$.

**Fig. 2:**
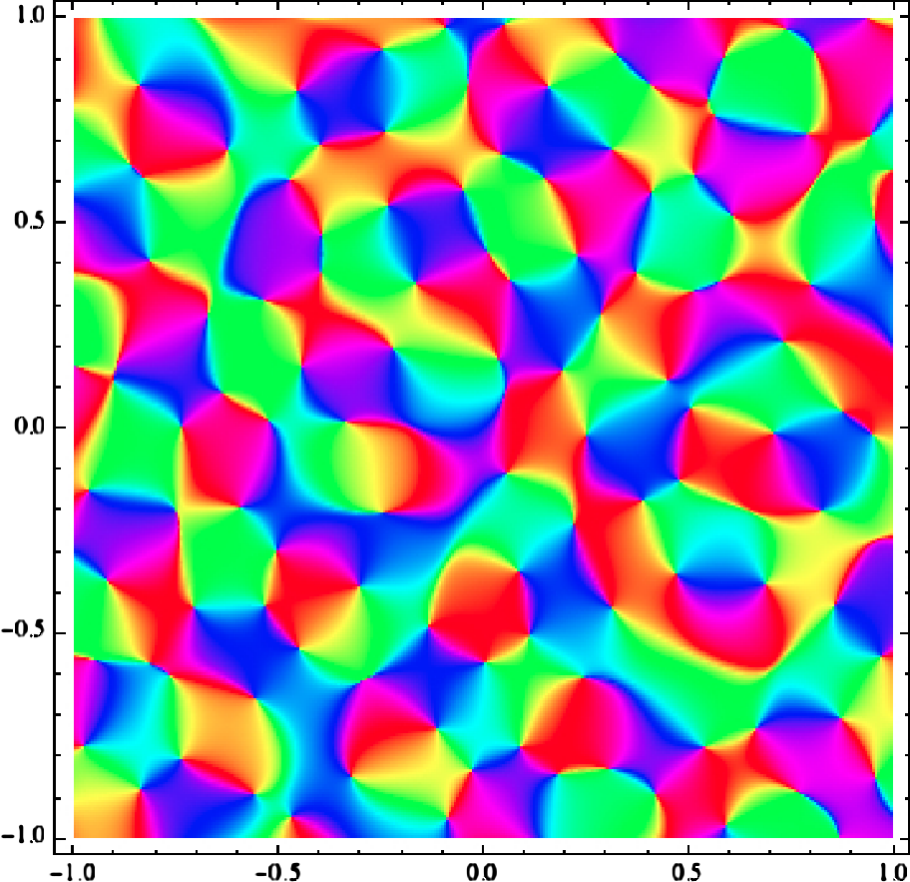
Computer-generated map, sampled from a monochromatic field. This figure shows an orientation map which we have drawn from a simulated invariant Gaussian random field with circular power spectrum. This figure was generated using a superposition of 30 plane waves with frequency vectors at the vertices of a random polygon inscribed in a circle, and random Gaussian weights (see the Appendix); what is plotted is the argument. In the unit of length displayed on the *x*- and *y*-axes, the wavelength is $1/3$ here

This determines a unique GRF **z** (see the Appendix for details on how to construct it from *Γ*), so let us draw an orientation map from this **z**: the result is shown on Fig. 2.

This looks realistic enough. Now, what is truly remarkable is that it is not only on a first, qualitative look that this map, which has been computer-generated from simple principles, has the right features: it also exhibits a pinwheel density of *π*, which Kaschube et al. have observed in real maps with 2 % precision [11].

Indeed, if $\mathcal {N}_{A}$ is the random variable recording the number of pinwheels in a region *A* with area $|A|$, it may be shown (using a formula of Kac–Rice type, see [38, 46] for background on the Kac–Rice formula) that

$$\mathbb{E} \biggl\{ \frac{\mathcal {N}_{A}}{|A|} \biggr\} = \frac{\pi }{\varLambda^{2}}. $$

This result appeared in physics [12, 26, 47] and is now supported by full mathematical rigor (see [48], Chap. 6, for the general setting and [49], Sect. 4 for the full proof); we shall use the same methods to derive non-Euclidean pinwheel densities in Sect. 3. We should note here that as translation-invariant random fields of our type have ergodicity properties (see [38], Sect. 6.5), it is quite reasonable to compare ensemble expectations for Gaussian fields and pinwheel densities which, in experiments, are measured on individual orientation maps.

Of course the correlation spectra measured in real V1 maps are not concentrated on an infinitely thin annulus (for precise measurements, see Schnabel [31], p. 103). But upon closer examination (see for instance [34]), one can see that maps sampled from invariant Gaussian fields whose spectra are not quite monochromatic, but concentrated on thin annuli, do not only look the same as that of Fig. 2, but that many interesting quantitative properties (such as pinwheel density or a low variance for the spacing between iso-orientation domains) have vanishing first-order terms as functions of spectral thickness. In other words, it is reasonable to say that monochromatic invariant random fields provide as good a description for the layout of orientation preferences as invariant fields with more realistic spectra do (perhaps even better; see [34]). As we shall see presently, neglecting details in the power spectrum and going for maximum simplicity allows for a generalization that will lead us to pinwheel-like arrangements in non-Euclidean settings. We shall take this step now and start looking for non-Euclidean analogues of monochromatic fields.

But to sum up, let us insist that three hypotheses introduced in [12] gave map ensembles with realistic qualitative and quantitative properties:

1. a *randomness structure*, that of a smooth Gaussian field;
2. an assumption of *Euclidean invariance*;
3. and a *monochromaticity, or near-to-monochromaticity condition* out of which quasi-periodicity in the map arose.

When we go over to non-Euclidean settings, these are the three properties that we shall look for. The first only needs the surface on which we draw orientation maps to be smooth. For analogues of the last two conditions in non-Euclidean geometries we need group actions, of course, and a non-Euclidean notion of monochromatic random field. In the next two subsections, we shall describe the appropriate tool.

Before we embark on our program, let us note that in spite of the close resemblance between maps sampled from monochromatic Gaussian fields and real mature maps, there are notable differences. As we remarked above, real correlation spectra are not infinitely thin, and the precise measurements by Schnabel make it possible to give quantitative arguments for the difference between an invariant Gaussian field with the measured spectrum (see for instance a discussion in [34]). In the successful long-range interaction model of Wolf, Geisel, Kaschube and coworkers, Gaussian fields turn out to be a better description of the initial stage of cortical map development than they are of the mature stage. We have two reasons for sticking to Gaussian fields in this paper: the first is that they are ideally suited to discussing and generalizing the concepts crucial to producing realistic maps, and the second is that a non-Euclidean version of the long-range interaction model can easily be written down in the upcoming Discussion.

### Klein Geometries

What is a space *M* in which conditions (1) and (2) have a meaning? Condition (1) says we should look for Gaussian fields whose trajectories yield smooth maps, so *M* should be a smooth manifold. To generalize condition (2), we need a group of transformations acting on *M*, with respect to which the invariance condition is to be formulated. Felix Klein famously insisted [50] that the geometry of a smooth manifold *M* on which there is a transitive group action is completely determined by a pair $(G, K)$ in which *G* is a Lie group and *K* a closed subgroup of *G*. We shall recall here some aspects of Klein’s view, focusing for the two-dimensional examples which we will use in the rest of this paper. This is famous and standard material; see the beautiful book by Sharpe [51], Chap. 4.

First, let us examine the previous construction and note that every geometrical entity we met can be defined in terms of the Euclidean group $\mathit{SE}(2) $. write *K* for the subgroup of rotations around a given point, say *o*. If $g = (R, \vec{x})$ is any element of *G*, the conjugate subgroup $gKg^{-1} = \{ (A, \vec{x} -A \vec{x}), A \in K \}$ is the set of rotations around $o+\vec{x}$. Now, the set $gK = \{ (A, x), A \in K \}$ remembers *x* and only *x*, so we can recover the Euclidean plane by considering the family of all such cosets, that is, the set $G/K = \{ gK, g \in G \}$.

Now when *G* is a general Lie group and *K* is a closed subgroup, the smooth manifold $M = G/K$ comes with a natural transitive *G*-action, and *K* is but the subset of transformations which do not move the point $\{K\}$ of *M*. This is summarized by saying that *M* is a *G*-homogeneous space.

*With this in mind, we can rephrase our main objective in this paper: it is to show that some Klein pairs*$(G, K)$*allow for a construction of V1-like maps on the homogeneous space*$M = G/K$*, and a calculation of pinwheel densities in these V1-like maps. We shall keepMtwo-dimensional here, and stick to the three maximally symmetric spaces* [52]*—the Euclidean plane, the round sphere and the hyperbolic plane.*

To recover the usual geometry of the round sphere $\mathbb{S}^{2}$ from a Klein pair, we need the group of rotations around the origin in $\mathbb {R}^{3}$, that is, $G = \mathit{SO}(3) = \{ A \in\mathcal{M}_{3}(\mathbb {R}) | ^{t}AA = I_{3} \text{ and } \det(A) = 1 \} $, and the closed subgroup $K = \{ \bigl({\scriptsize\begin{matrix}R & 0\cr 0 & 1 \end{matrix}}\bigr) , R \in \mathit{SO}(2) \}$—of course *K* is the group of rotations fixing $(0,0,1)$.

Let us now briefly give some details of how the hyperbolic plane can be defined from a Klein pair. Here *G* is the group of linear transformations of $\mathbb {C}^{2}$ that have unit determinant and preserve the quadratic form $(z, z') \mapsto|z|^{2} -|z'|^{2}$, that is,

$$G = \mathit{SU}(1,1) = \left\{ \begin{pmatrix} \alpha& \beta\\ \bar{\beta} & \bar{\alpha} \end{pmatrix} \Big| \alpha, \beta\in \mathbb {C}, |\alpha|^{2} -|\beta|^{2} = 1 \right\}. $$

Elements of *G* operate on the complex plane $\mathbb {C}$ via conformal (but nonlinear) transformations: any element $g= \bigl({\scriptsize\begin{matrix} \alpha& \bar{\beta} \cr \beta& \bar{\alpha} \end{matrix}}\bigr) $ of *G* gives rise to a homography $z \mapsto g \cdot z:= \frac{\alpha z + \beta}{\bar{\beta} z + \bar{\alpha}}$ of the complex plane. It is easy to see that the origin can be sent anywhere on the *(open) unit disk*, but nowhere outside. Now the subgroup *K* of transformations that leave 0 invariant is obviously $K = \{ \bigl({\scriptsize\begin{matrix} e^{i\varphi} & 0 \cr 0 & e^{-i \varphi} \end{matrix}}\bigr) , \phi\in \mathbb {R}\}$; note that its elements induce the ordinary rotations of the unit disk. So the unit disk $\mathbb{D}$ in $\mathbb {C}$ comes with a Klein pair $(G,K)$, and looking for a *G*-invariant metric on $\mathbb{D}$ famously produces the negatively-curved *Poincaré metric* (see [43, 53] and the Appendix for details). Recall that the formula for the square of the length of a vector tangent to $\mathbb{D}$ at $(x,y)$ is summarized by

$$ds^{2} = \frac{4}{(1-(x^{2}+y^{2})^{2})^{2}}\bigl(dx^{2} + dy^{2} \bigr). $$

We shall write $\eta(p)$ for the numerical function $p = (x,y) \mapsto \frac{4}{(1-(x^{2}+y^{2})^{2})^{2}}$, and ***η*** for the abstract *G*-invariant metric we have just defined. Of course group theory allows for a simple description of all its geodesics and of many other things geometrical, and we shall need those: to keep the size of the present section reasonable, we delay this description to Sect. 2.1.

But now let us go forward to meet one of the many reasons why Klein’s description of spherical and hyperbolic geometries, far from being a matter of aesthetics, shows our concrete tools of map engineering the way to non-Euclidean places.

### Group Representations and Noncommutative Harmonic Analysis

#### Unitary Representations

Assume we are given one of the two non-Euclidean Klein pairs $(G, K)$ above and we wish to build an orientation map with properties (1), (2), and (3) from Sect. 2.2. Conditions (1) and (2) say we should use a smooth complex-valued Gaussian random field that is invariant under the action of *G*. We shall come back to exploiting condition (2) in time. But condition (3) depends on classical Fourier analysis, which is based using plane waves and thus seems tied to $\mathbb {R}^{n}$.

Fortunately there *is* a completely group-theoretical description of classical Fourier analysis too: for details, we refer to the beautiful survey by Mackey [54]. One of its starting points is the fact that for functions defined on $\mathbb {R}^{n}$, the Fourier transform turns a global translation of the variable (that is, passage from a function *f* to the function $x \mapsto f(x - x_{0})$) into multiplication by a universal (nonconstant) factor (the Fourier transform $\widehat{f}$ is turned into $k \mapsto e^{i k x_{0}} \widehat {f}(k)$). From this behaviour of the Fourier transform under the *action of the group of translations*, some of those properties in Fourier analysis which are wonderful for engineering—like the formula for the Fourier transform of a derivative—follow immediately.

For many groups, including $\mathit{SO}(3)$ and $\mathit{SU}(1,1)$ which we will use in this paper, there is a “generalized Fourier transform” which gives rise to analogues of the property we just emphasized, although it is technically more sophisticated than classical Fourier analysis. It is best suited to analyzing functions defined on spaces with a *G*-action, yielding concepts of “generalized frequencies” appropriate to the group *G* [55, 56].

It will then come as no surprise that the vocabulary of noncommutative harmonic analysis is well suited to describing the invariant Gaussian field model for orientation preference maps in V1, since the key features of this model rest on the action of $\mathit{SE}(2) $ on the function space of orientation maps. As soon as we give details, it will also be apparent that an analogue of the monochromaticity condition (3) can be formulated in terms of these “generalized frequencies”.

Before we discuss its significance and its relevance to Euclidean (and non-Euclidean) orientation maps, we must set up the stage for harmonic analysis; so we beg our reader for a little mathematical patience until Sect. 2.4.3 brings us back to orientation maps.

Let *G* be a Lie group. Representation theory starts with two definitions: a *unitary representation ofG* is a continuous homomorphism, say *T*, from *G* to the group $\mathcal{U}(\mathcal{H})$ of linear isometries of a Hilbert space; we write $(\mathcal{H}, T)$ for it. This representation is *irreducible* when there is no $T(G)$-invariant closed subspace of $\mathcal{H}$ except $\{0\}$ and $\mathcal{H}$.

We need to give two essential examples, the second of which is crucial to the strategy of this paper:

1. If *p* is a vector in $\mathbb {R}^{n}$, define $T_{p}(x) = e^{i p \cdot x}$ for each $x \in G = \mathbb {R}^{n}$; this defines a continuous morphism from $G = \mathbb {R}^{n}$ to the unit circle $\mathbb {S}^{1}$ in $\mathbb {C}$; by identifying this unit circle with the set of rotations of the complex line $\mathbb {C}$, we may say that $(\mathbb {C}, T_{p})$ is an irreducible, unitary representation of $\mathbb {R}^{n}$. In fact, every irreducible unitary representation of $\mathbb {R}^{n}$ reads $(\mathbb {C}, T_{p})$ where *p* is a vector. Thus, the set of irreducible representations of the group of translations on the real line or of an *n*-dimensional vector space is nothing else than the set of “time” or “space” frequencies in the usual sense of the word.^4^
2. Suppose *M* is the real line, the Euclidean plane, the sphere or the hyperbolic plane, and *G* the corresponding isometry group. If *f* is a complex-valued function on *M*, define $L(g) f:= x \mapsto f(g^{-1} \cdot x)$. Then for every $g \in G$, $L(g)$ defines a unitary operator acting in the Hilbert space $\mathbb{L}^{2}(M)$ (here integration is with respect to the measure determined by the metric we chose on *M*); so we get a canonical unitary representation $(\mathbb {L}^{2}(M), \mathcal {L})$ of *G*. It is very important to note that this representation is *reducible* in our four cases; we discuss its invariant subspaces in the next subsection.

A word of caution: our first example, although it is crucial to understanding how representation theory generalizes Fourier analysis, is much too simple to give an idea of what *irreducible* representations of nonabelian groups are like; for instance, the space $\mathcal{H}$ of an irreducible representation very often happens to be infinite-dimensional (the first and most famous examples are in [57]), and this will be crucial in our discussion of hyperbolic geometry.

#### Plancherel Decomposition

Suppose *M* is the Euclidean plane, the hyperbolic plane or the sphere. We shall now give an outline of the *Plancherel decomposition of*$\mathbb{L}^{2}(M)$, which is crucial to our strategy for producing non-Euclidean orientation maps. This is standard material: for details, we refer to [53], Chap. 0.

Let us consider the representation $\mathcal {L}$ of example 2 above, acting on $\mathcal{H} = \mathbb{L}^{2}(M)$. Since is not irreducible, we may write $\mathcal{H} = \mathcal{H}_{a} \oplus\mathcal{H}_{b}$ where $\mathcal{H}_{a}$ and $\mathcal{H}_{b}$ are mutually orthogonal, *stable* subspaces of $\mathcal{H}$ (note that for this to be so, they must be closed), and try to decompose $\mathcal{H}_{a}$ further. We may hope to come to a *decomposition into irreducibles*, and hope to eventually be able to write

$${\mathcal{H} = \bigoplus_{\gamma} \Biggl( \bigoplus _{i = 1}^{m_{\gamma}} \mathcal{H}_{\gamma,i} \Biggr)}, $$

a direct sum of invariant, mutually orthogonal subspaces $\mathcal {H}_{\gamma, i}$ which inherit *irreducible* representations of *G* from $\mathcal {L}$, with $\mathcal{H}_{\gamma, i}$ equivalent^5^ to $\mathcal{H}_{\gamma', i'}$ if and only if $\gamma= \gamma'$.

When *G* is the rotation group $\mathit{SO}(3)$ and *M* is the sphere, or more generally when *G* is compact, this is actually what happens, and it is a part of the Peter–Weyl theorem that the above direct sum decomposition holds. In the case of the sphere, all the $m_{\gamma}$s will be equal to 1 and we will describe the $\mathcal{H}_{\gamma}$ in Sect. 3.2.1. But for the noncompact groups $\mathit{SE}(2)$ and $\mathit{SU}(1,1)$, the decomposition process turns out to degenerate.

A simpler example will help us understand the situation: consider the representation $\mathcal {L}$ of $\mathbb {R}$ on $\mathbb{L}^{2}(\mathbb {R})$ (example 2 above). Since a change of origin induces but a (nonconstant) phase shift in the Fourier transform, the subspace $\mathcal{F}_{I}$ of functions whose Fourier transform has support in interval *I*, is invariant by each of the $\mathcal {R}(x)$, $x \in \mathbb {R}$. But now it is true also that, say, $\mathcal{F}_{[0,1]} = \mathcal{F}_{[2,2.5]} \oplus \mathcal{F}_{[2.5, 3]} = \mathcal{F}_{[2,2.25]} \oplus\mathcal{F}_{[2.25,2.5]} \oplus\mathcal{F}_{[2.5,2.75]} \oplus\mathcal{F}_{[2.75,3]}$, and so on. Since we can proceed to make the intervals smaller and smaller, we see that an irreducible subspace should be a one-dimensional space of functions which have only one nonzero Fourier coefficient, in other words, each member of the irreducible subspaces should be a plane wave… which is *not* a square-integrable function! So in this case, there is *no* invariant subspace of $\mathbb{L}^{2}(\mathbb {R})$ that inherits an irreducible representation from $\mathcal {R}$, and it is only by getting out of the original Hilbert space that we can identify irreducible “constituents” for $\mathbb {L}^{2}(\mathbb {R})$.

When *M* is the Euclidean plane or the hyperbolic plane, this is what will happen: starting from $\mathbb{L}^{2}(M)$, we shall meet spaces $\mathcal{E}_{\omega}$ of *smooth* (and a priori not square-integrable) functions which

- are invariant under the canonical operators $\mathcal {L}(g)$, $g \in G$,
- carry irreducible unitary representations of *G*,
- and together give rise to the following version of the Plancherel formula: for each $f \in\mathbb{L}^{2}(M)$ and for almost every *x* in *M*,
  $$f(x) = \int_{\omega\in\mathcal{F}} f_{\omega}(x)\, d\varPi(\omega), $$
  where $\mathcal{F}$ is some set of equivalence classes of representations of *G* (the “frequencies”), *Π* is a measure on $\mathcal{F}$ (the “power spectrum”), and for each $\omega\in\mathcal {F}$, $f_{\omega}$ is a member of $\mathcal{E}_{\omega}$ (a smooth function, then).

Recall that our aim in introducing noncommutative harmonic analysis is to find an analogue of the monochromaticity condition (3), Sect. 2.2, in spherical and hyperbolic geometry. As we shall see presently, the situation in the Euclidean plane makes it reasonable to call an element of $\mathcal{E}_{\omega}$ or $\mathcal{H}_{\gamma}$ a *monochromatic map*. Belonging to one of the $\mathcal{E}_{\omega}$, resp. one of the $\mathcal{H}_{\gamma}$, will be our non-Euclidean analogue of the monochromaticity condition (3) in hyperbolic geometry, resp. spherical geometry. We shall see that a Gaussian random field providing orientation-preference-like maps may be associated to each of these spaces of monochromatic maps, and that it yields quasi-periodic tilings of *M* with Euclidean-like pinwheel structures.

#### Relationship with Euclidean Symmetry in V1

Let us now proceed to relate the Plancherel decomposition of $\mathbb{L}^{2}(\mathbb {R}^{2})$ to the monochromaticity condition (3) in Sect. 2.2. In the notations of Sect. 2.2, (3) means that the correlation function *Γ* of a monochromatic field should have its support on the circle of radius $\frac{2\pi}{\varLambda}$, hence satisfy the Helmholz equation

(3′): $\Delta\varGamma= - ( \frac{2\pi}{\varLambda} )^{2} \varGamma $

and we already pointed out that adding rotation invariance (and normalizing $\varGamma(0)$ to be 1) determines *Γ* to be the Dirac distribution on the circle of radius $\frac{2\pi}{\varLambda}$. Now the space $\mathcal {E}_{\varLambda}$ of all smooth maps *φ* satisfying $\Delta\varphi= - ( \frac{2\pi}{\varLambda} )^{2} \varphi$ has the following properties:

- if *φ* is in $\mathcal {E}_{\varLambda}$, then $g\cdot\varphi : x \mapsto\varphi(g^{-1} x)$ is in $\mathcal {E}_{\varLambda}$ for any $g \in E(2)$; this means $\mathcal {E}_{\varLambda}$ is an invariant subspace of the set of smooth maps;
- $\mathcal {E}_{\varLambda}$ has itself no closed invariant subspace if one uses the usual smooth topology for it: indeed if *φ* is any nonzero element in $\mathcal {E}_{\varLambda}$, it may be shown that the family of maps $g\cdot\varphi$, $g \in G$, generates a dense subspace of $\mathcal {E}_{\varLambda}$. Perhaps a word of caution is useful here: while *Γ* is rotation-invariant and determines a *G*-invariant random field, it is certainly not itself invariant under the full group *G* of motions.

Let us insist that condition (3′) may now be rewritten as:

(3″): *Γ* belongs to one of the elementary invariant subspaces $\mathcal {E}_{\varLambda}$.

Now, suppose we start with any square-integrable map *f* from $\mathbb {R}^{2}$ to $\mathbb {C}$ with continuous Fourier transform; for each $K>0$, we may restrict $\widehat{f}$ to the circle of radius *K* to produce the map

$$f_{K} = \vec{x} \mapsto\int_{\mathbb {S}^{1}} \widehat{f}(K \vec{u})e^{{i} K\vec {u} \cdot\vec{x}} \,d\vec{u} $$

which is automatically smooth, but not square-integrable;^6^ see [58] for details. And then for almost every *x*,

$$f(x)= \int_{\mathbb {R}^{+}} f_{K}(x) K\,dK. $$

This shows that the $\mathcal{E}_{\varLambda}$ do provide the factors in the Plancherel decomposition of $\mathbb{L}^{2}(\mathbb {R}^{2})$ described at the end of Sect. 2.4.2, and the equivalence between conditions (3) and (3″) shows how the spectral thinness condition found in models is related to the Plancherel decomposition of $\mathbb{L}^{2}(\mathbb {R}^{2})$. In Sect. 2.2, we saw how each of this factors determines a unique Gaussian random field which provides realistic V1-like maps.

We now have gathered all the ingredients for building two-dimensional V1-like maps with non-Euclidean symmetries. But before we leave the Euclidean setting, let us remark that the irreducible representation of $\mathit{SE}(2)$ carried by the $\mathcal{E}_{\varLambda}$ has been used in [39], although the presentation there is rather different.^7^ While the approach of [39], which brings the horizontal connectivity to the fore and uses Heisenberg’s uncertainty principle to exploit the noncommutativity of $\mathit{SE}(2)$, has notable differences with using Gaussian random fields, it is very interesting and defines *real*-valued random fields which are good candidates for the maps $a_{\vartheta}$ of Sect. 2.1. To the author’s knowledge this is the first time irreducible representations of $\mathit{SE}(2)$ were explicitly used to study V1, and reading this paper was the starting point for the present study.

## Results

### Hyperbolic Geometry

Let us now turn to plane hyperbolic geometry. The relevant groups for capturing the global properties of the hyperbolic plane assemble in the Klein pair $(G,K) = (\mathit{SU}(1,1), \mathit{SO}(2))$ as described in Sect. 2.

If we are to look for pinwheel-like arrangements lurking in the representation theory of $\mathit{SU}(1,1)$, we need a familiarity with some irreducible representations. We shall use the next paragraph to give the necessary details on the geometry of the unit disk; the description of all unitary representations of $\mathit{SU}(1,1)$, however, we shall skip over^8^ in order to focus on the Plancherel decomposition of $\mathbb{L}^{2}(G/K)$.

We should note at this point that hyperbolic geometry and $SL_{2}$-invariance^9^ have been used by Chossat and Faugeras for a different purpose [59]; the same basic ingredients will appear here.

#### Geometrical Preliminaries

In this subsection, we must ask again for a little mathematical patience from our reader while we introduce some geometrical notions which we shall need for building hyperbolic maps (this is very standard material again; see [53], Sect. 0.4, and the paper by Chossat and Faugeras). So let us first describe some further interplay between the algebraic structure of $G=\mathit{SU}(1,1)$ and hyperbolic geometry in the unit disk. Geodesics in $\mathbb{D}$ are easily described in terms of groups: since the action of *G* leaves the metric ***η*** invariant, the energy functional whose extremal paths are the geodesics of $\mathbb{D}$ is *G*-invariant as well; so any element $g \in G$ sends geodesics to geodesics. What is more, the horizontal path $t \mapsto\gamma(t) = (\operatorname{tanh}(t), 0)$ has hyperbolic unit speed and it is not difficult to show that it is a geodesic of $\mathbb{D}$ (see [53], p. 29). Now, the interplay between group theory and Riemannian geometry makes it easy to find all geodesics of $\mathbb {D}$. Since $\gamma(t)$ is where the origin is sent by the element $\bigl({\scriptsize\begin{matrix} \cosh(t) & \sinh(t) \cr \sinh(t) & \cosh(t) \end{matrix}}\bigr) $ of *G*, there is a subgroup of *G* to tell the story of the point 0 along this path: it is the subgroup

$$A = \left\{ \begin{pmatrix} \cosh(t) & \sinh(t) \\ \sinh(t) & \cosh(t) \end{pmatrix} \Big| t \in \mathbb {R}\right\}. $$

From conjugates of this subgroup we may describe all geodesics in $\mathbb{D}$: if we start with a point $x_{0}$ of $\mathbb{D}$ and a tangent vector $v_{0}$ at $x_{0}$, there is an element *g* of *G* which sends both $x_{0}$ to the origin and $v_{0}$ to the right-pointing horizontal unit vector. But now the geodesic emanating from $x_{0}$ with speed $v_{0}$ is none other than the orbit of $x_{0}$ under the subgroup $g^{-1}Ag$; as *g* acts through a homography on $\mathbb{D}$, it is easy to see that this orbit draws on $\mathbb {D}$ a circle that is tangent to $x_{0} + \mathbb {R}v_{0}$ and orthogonal to the boundary of $\mathbb{D}$ (this “circle” might be a line, which we can think of as a circle of infinite radius here).

Just as a family of parallel lines in $\mathbb {R}^{2}$ has an associated family of parallel hyperplanes that are orthogonal to each line in the family, the set of *A*-orbits has an associated family of *parallel horocycles*: writing $b_{0}$ for the point of the boundary $\partial \mathbb{D}$ that is in the closure of every *A*-orbit (i.e. the point $1+0i$ in $B = \partial\mathbb{D}$), a circle that is tangent to $\partial\mathbb{D}$ at $b_{0}$ meets every *A*-orbit orthogonally. What is more, given two such circles, there is on any *A*-orbit a unique segment that meets them both orthogonally; the length of this hyperbolic geodesic segment does not depend on the *A*-orbit chosen, so it is very reasonable indeed to call our two circles parallel. Circles tangent to $\partial\mathbb{D}$ were named *horocycles* by Poincaré, so we have been looking at the (parallel) family of those horocycles that are tangent to $\partial\mathbb{D}$ at $b_{0}$.

Now these horocycles too come with a group to tell their tale: they are the orbits in $\mathbb{D}$ of

$$N = \left\{ \begin{pmatrix} is & 1 -is \\ -is & is \end{pmatrix} \Big| s \in \mathbb {R}\right\}. $$

To describe the families of parallel horocycles associated to other families of geodesics it is a conjugate of *N* that should be used: for this we should first note that if *g* is any element of the group, the family of $g^{-1} N g$-orbits consists of horocycles tangent to $\partial\mathbb{D}$ at the same point, and then that each of these horocycles meets every $g^{-1} A g$-orbit orthogonally.

There is one more definition that we shall need: it is closely linked to an important theorem in the structure of semisimple Lie groups [52, 60].

##### Theorem

*Every element*$g \in G = \mathit{SU}(1,1)$*may be written uniquely as a product*^10^$k a n$, *where*$k \in K$, $a \in A$, *and*$n \in N$. *This is known as an* Iwasawa decomposition *forG*.

Note that *K*, *A*, and *N* are one-dimensional subgroups of *G*, but that the existence of a unique factorization $G = KAN$ does not mean at all that *G* is isomorphic with the direct product of *K*, *A*, and *N*.

Even if this is a very famous result, an idea of the proof will be useful for us. Note first that any point $x \in\mathbb{D}$ may be reached from *O* by following the horizontal geodesic for a while (forwards or backwards) until one reaches the point of the horizontal axis which is on the same horocycle in the family of *N*-orbits, then going for *x* along this circle; this means that we can write $x = n\cdot (a \cdot O)$, where $n \in N$ and $a \in A$; now if *g* is any element of *G*, we may consider $x = g \cdot O$ and write it $x = n \cdot(a \cdot O) = (na) \cdot O$; then $(na)^{-1}g$ sends *O* to *O*, so it is an element of *K*. This proves the existence statement; uniqueness is easy but more technical.

Now if *b* is a point of the boundary $\mathbb{D}$ that has principal argument *θ*, we may view it as an element of *K* by assigning to it the element $\bigl({\scriptsize\begin{matrix} e^{i \theta} & 0 \cr 0 & e^{-i\theta} \end{matrix}}\bigr) $; note that this element acts on $\mathbb{D}$ as a rotation of angle 2*θ*! So beware, diametrally opposite elements *b* and −*b* of the boundary define the same rotation.

If *x* is any point of $\mathbb{D}$ and *b* is any boundary point, we can now define a real number $\langle x,b \rangle$ as follows: start with any element $\tilde{x}$ of *G* that sends *x* to *O*, then choose a representative $\tilde{b}$ of *b* in *K* and consider the Iwasawa decomposition of the element $\tilde{x} \cdot\tilde{b}$ of *G*: it reads

$$\tilde{x} \cdot\tilde{b} = k a n. $$

Now look at *a*, and consider the real number *t* such that $a = \bigl({\scriptsize\begin{matrix} \cosh(t) & \sinh(t) \cr \sinh(t) & \cosh(t) \end{matrix}}\bigr) $. Write $\langle x,b \rangle$ for this number *t*; it is not difficult to check that this does not depend on any of the choices one has to make to select $\tilde{x}$ or $\tilde{b}$.

**Fig. 3:**
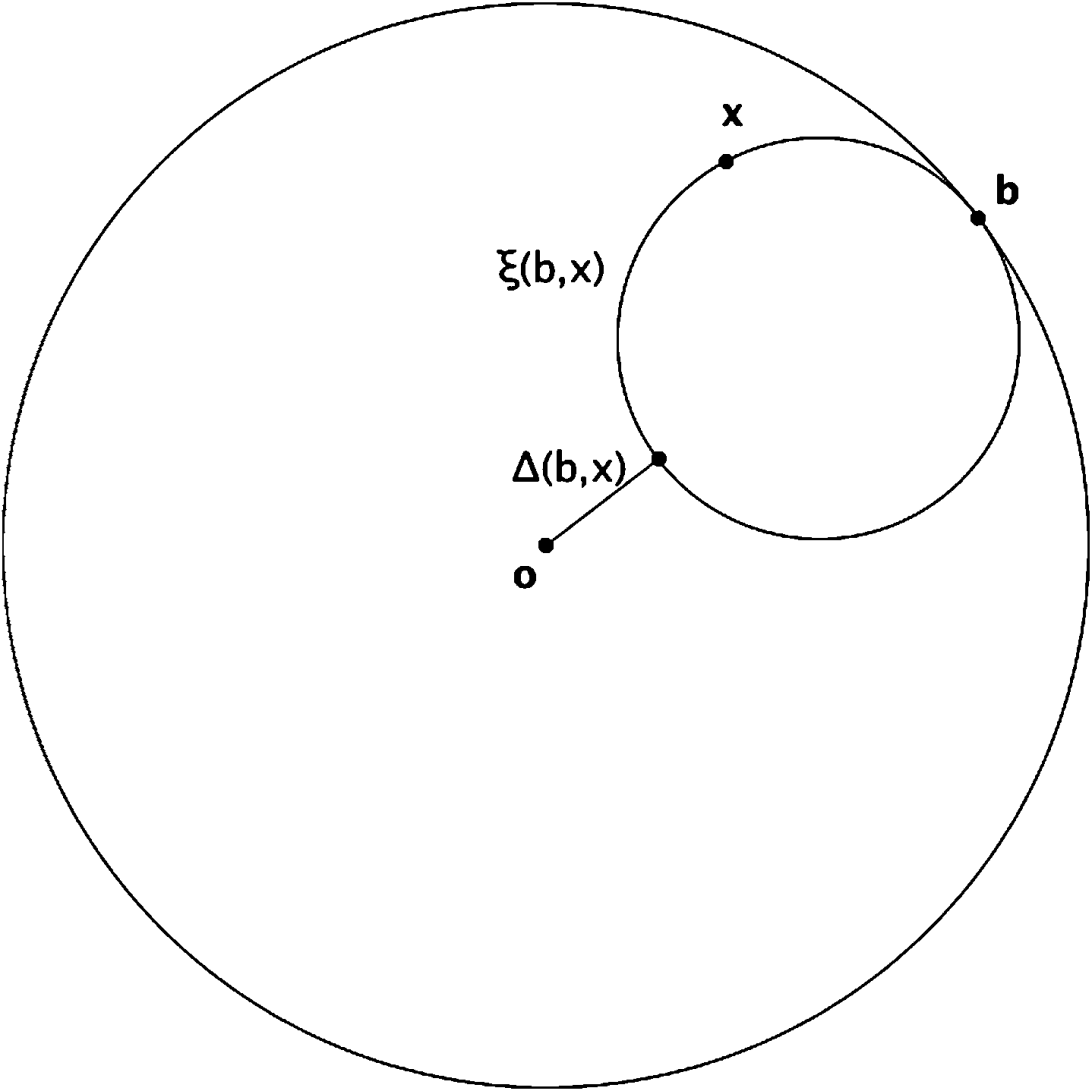
The “composite distance” to a point of the boundary. Definition of the quantity $\langle x, b \rangle$ if *x* is a point of $\mathbb{D}$ and *b* a point of its boundary: $\xi(b,x)$ is the horocycle through *x* which is tangent to the boundary at *b*, and $\Delta(b, x)$ is the segment joining the origin *O* to the point on $\xi(b,x)$ which is diametrically opposite *b*; the number $\langle x, b \rangle$ is, up to a sign, the hyperbolic length of this segment

The indications we gave for the proof of the Iwasawa decomposition led Helgason to call $\langle x,b \rangle$ a (signed) *composite distance*; the definition and its relationship with the hyperbolic distance are illustrated on Fig. 3.

#### Helgason Waves and Harmonic Analysis

At first sight, there is no reason why harmonic analysis on the hyperbolic plane should “look like” Euclidean harmonic analysis: their invariance groups are apparently quite different and there is nothing like an abelian “hyperbolic translation group” whose characters may obviously be taken as a basis for building representation theory. So it may come as a surprise that there *are* analogues of plane waves in hyperbolic space, and (more importantly) that these enjoy much the same relationship to hyperbolic harmonic analysis as Fourier components do to Euclidean analysis. The discovery of these plane waves can be traced back to the seminal work of Harish-Chandra [61] on spherical functions of semi simple Lie groups (we shall come back to this in a moment), and their systematic use in non-Euclidean harmonic analysis is due to Helgason [53, 62]. Since they will be a key ingredient in the rest of this section, let us now describe these waves.

Start with a point *b* of the boundary $B:= \partial\mathbb{D}$ and a real number *ω*. For ${z} \in\mathbb{D}$, set

$$e_{\omega,b}(z):= e^{(i\omega+ 1)\langle z , b\rangle}. $$

**Fig. 4:**
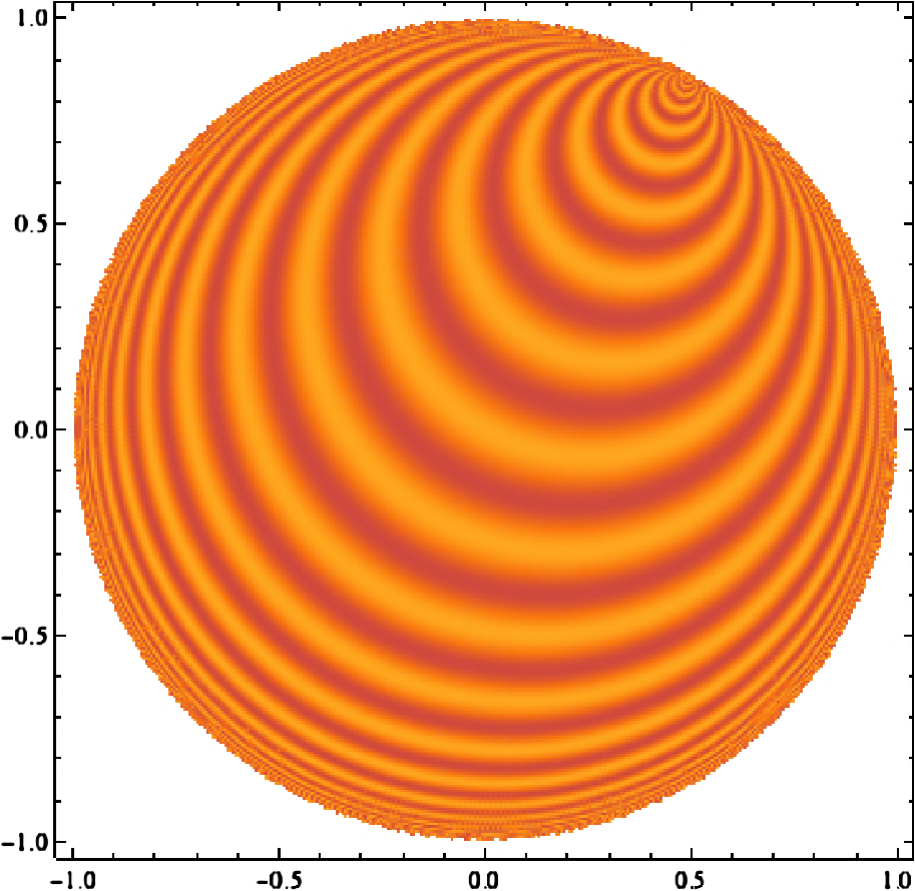
Plot of the real part of a Helgason wave, with the exponential growth factor deleted: in the darkest regions the real part of the scaled wave vanishes, and in the brightest regions it is equal to one. Given the formula for $e_{\omega,b}$, this plot also gives an idea of the argument as a function of *z*; notice that the argument is periodic when restricted to any geodesic whose closure in $\mathbb {C}$ contains the point of −1 of *B*

This is a complex-valued function on $\mathbb{D}$; note that as *z* draws close to *b*, $\langle z,b \rangle$ goes to infinity, so $e_{\omega,b}(z)$ grows exponentially; on the other hand it decreases exponentially as *z* draws close to −*b*. This growth factor in the modulus is there for technical reasons, but has important consequences for representation theory and in the case of our orientation maps, it will have a clear influence on the pinwheel density we shall calculate later; we shall discuss this at the end of the present section. For a plot indicating the argument of $e_{\omega,b}$; see Fig. 4.

Just as plane waves are generalized eigenvectors for the Euclidean Laplacian on $\mathbb {R}^{2}$, Helgason waves are “eigenvectors” for the relevant Laplacian. Define, for $\mathcal{C}^{2}$*f*,

$$\Delta_{\mathbb{D}} f:= p \mapsto{\frac{1}{\eta(p)}}( \Delta_{\mathbb {R}^{2}} f) (p). $$

This is indeed the Laplace operator for $\mathbb{D}$: it can be defined from group-theoretical analysis alone, in much the same way we obtained the Poincaré metric in Sect. 2.3 and the Appendix. Theoretical questions aside, the reader may check easily that this new Laplacian is *G*-invariant, that is, $\Delta_{\mathbb{D}} [ f(g^{-1} \cdot) ] = [ \Delta_{\mathbb{D}} f ](g^{-1} \cdot)$.

Now, a crucial observation is that $e_{\lambda,b}$ is an eigenfunction for this operator, with a *real* eigenvalue:^11^

$$\Delta_{\mathbb{D}} e_{\lambda,b} = -\bigl(\omega^{2} + 1\bigr) e_{\lambda,b}. $$

As a consequence, any finite combination of the $e_{\omega,b}$ with *ω* fixed is an “eigenvector” for $\Delta_{\mathbb{D}}$. Now let us go for continuous combinations: if $\mu: B \rightarrow \mathbb {R}$ is a continuous function, then

$$\mathcal{P}_{\omega}(\mu):= z \mapsto\int_{B} e_{\omega,b}(z) \mu (b) \,db $$

(the *Poisson transform* of *μ*) is another eigenfunction with eigenvalue $-(\omega^{2} + 1)$:

$$\Delta_{\mathbb{D}} \bigl[\mathcal{P}_{\omega}(\mu)\bigr] = -\bigl( \omega^{2} + 1\bigr)\bigl[\mathcal{P}_{\omega}(\mu)\bigr]. $$

We shall write $\mathcal{E}_{\omega}(\mathbb{D})$ for the space of all such smooth eigenfunctions:

$$\mathcal{E}_{\omega}(\mathbb{D}):= \bigl\{ f \in\mathcal{C}^{\infty}(\mathbb{D}, \mathbb {C}) | \Delta_{\mathbb{D}} f = -\bigl(\omega^{2} + 1 \bigr) f \bigr\} . $$

For each continuous function $\mu: B \rightarrow \mathbb {R}$, we then know that $\mathcal{P}_{\omega}(\mu)$ belongs to $\mathcal{E}_{\omega }(\mathbb{D})$, and in fact the image of $\mathcal{P}_{\omega}$ is dense in $\mathcal{E}_{\omega}(\mathbb{D})$ for several natural topologies (see [53], Chap. 0, Theorem 4.3, Lemma 4.20). Since $\Delta_{\mathbb{D}}$ is *G*-invariant, $\mathcal{E}_{\omega}(\mathbb {D})$ is a stable subspace of $\mathcal{C}^{\infty}(\mathbb{D})$; by studying $\mathcal{P}_{\omega}$, Helgason was able to prove that the $\mathcal{E}_{\omega}(\mathbb{D})$ is irreducible ([53], Chap. 0, Theorem 4.4). The following theorem then achieves the Plancherel decomposition of $\mathbb{L}^{2}(\mathbb{D})$ in the sense of Sect. 2.4.2, and is a cornerstone of harmonic analysis on the unit disk (see [53], Chap. 0, Theorem 4.2; the extension to $\mathbb{L}^{2}$ is proved there also):

##### Theorem

(Harish-Chandra, Helgason)

*For each*$f \in\mathcal {C}^{\infty}(\mathbb{D})$, *write*$f_{\omega}(z) = \int_{B} ( \int_{\mathbb{D}} f(y) e_{-\omega, b}(y)\,dy ) e_{\omega,b}(z) \,db$, *and set*$\varPi(\omega) = \frac{\omega}{2} \tanh(\frac{\pi\omega}{2})$*for each positiveω*; *then the following equality holds as soon as all terms are defined by converging integrals*:

$$f(z) = \int_{\mathbb {R}^{+}} f_{\omega}(z) \varPi(\omega)\, d\omega. $$

When we build our hyperbolic maps in the next section, the $\mathcal {E}_{\omega}(\mathbb{D})$ are the only representations we shall need. We will come back to this shortly.

#### Hyperbolic Orientation Maps

It is time to build our hyperbolic analogue of Orientation Preference Maps. Suppose we wish to arrange sensors on $\mathbb{D}$ so that each point of $\mathbb{D}$ is equipped with a receptive profile which has an orientation preference and a selectivity. This may be local model for an arrangement of V1-like receptive profiles on a negatively curved region of the cortical surface, and though its primary interest is probably in clarifying the role of symmetries in discussions, the construction to come can be thought of in this way.

We shall require that this arrangement have the same randomness structure (condition (1)) as the Euclidean model of Sect. 2, that is, be a “typical” realization of a standard complex-valued Gaussian random field on the space $\mathbb{D}$, say **z**. If it is to have an analogous invariance structure (conditions (2) and (3)), it should, first, be assumed to be G-invariant; what is more, we should look for a field that probes an irreducible factor of the representation of *G* on $\mathbb{L}^{2}(\mathbb {D})$ (see Sect. 2.4.2); as a consequence, any realization of **z** should be an eigenfunction of $\Delta_{\mathbb{D}}$, with the eigenvalue determined by **z**. Remembering the Euclidean terminology we used in Sect. 2, let us introduce the following notion.

##### Definition

*A monochromatic Gaussian field on*$\mathbb {D}$ is a complex-valued Gaussian random field on $\mathbb{D}$ whose probability distribution is $\mathit{SU}(1,1)$-invariant and which takes values in one of the $\mathcal{E}_{\omega}$, $\omega>0$. If **z** is such a field, the positive number *ω* will be called the spectral parameter of **z**.

To see how to build such a monochromatic field, we should translate our requirements into a statement about its covariance function; luckily there is a theorem here ([63], see the discussion surrounding Theorem 6′ and Theorem 7, in particular Eq. (3.20) there) that says our conditions on **z** are fulfilled if, and only if, the covariance function of **z**, when turned thanks to the *G*-invariance of **z** into a function from $\mathbb{D}$ to $\mathbb {C}$, is an elementary spherical function for $\mathbb{D}$ (a radial function on $\mathbb{D}$ which is an eigenfunction $\Delta_{\mathbb{D}}$). What does this mean?

First, note that the covariance function $C: \mathbb{D}^{2} \rightarrow \mathbb {C}$ of our field may be seen as a function $\tilde{C} = G^{2} \rightarrow \mathbb {C}$: we need only set $\tilde{C}(g_{1}, g_{2}) = C(g_{1} \cdot O, g_{2} \cdot O)$. Now, that **z** should be *G*-invariant means that for every $g_{0} \in G$, $\tilde{C}(g g_{1}, g g_{2})$ should be equal to $C(g_{1}, g_{2})$; in particular, writing $\varGamma(g)$ for $\tilde{C}(g, 1_{G})$, we get $\tilde{C}(g_{1}, g_{2}) = \varGamma(g_{2}^{-1} \cdot g_{1})$. The whole of the correlation structure of the field is summed up in this *Γ*, which is a function from *G* to $\mathbb {C}$.

Now, not every function from *G* to $\mathbb {C}$ can be obtained in this way: since it should come from a function *C* which is defined on $\mathbb {D}^{2}$ and thus satisfies $C(g_{1}k_{1},g_{2}k_{2}) = C(g_{1}, g_{2})$ when $k_{1}$, $k_{2}$ is in *K*, it should certainly satisfy $\varGamma(k_{1} g k_{2}) = \varGamma(g)$ for $k_{1}, k_{2} \in K$; so *Γ* does in fact define a function on $\mathbb{D}$ and this function is left-*K*-invariant, that is, radial in the usual sense of the word (*property* (A)). What is more, since the field is assumed to have variance 1 everywhere, it should also satisfy (B) $\varGamma(\mathrm{Id}_{G}) =1$ (*property* (B)).

Let us now add that monochromaticity for **z** is equivalent to *Γ* being an eigenfunction of $\Delta_{\mathbb{D}}$ (*property* (C)).

Functions on $\mathbb{D}$ with properties (A), (B), and (C) are called elementary spherical functions for $\mathbb{D}$.

Now, we stumbled upon these (following Yaglom) while looking for pinwheel-like structures, but spherical functions (and their generalizations to semisimple symmetric spaces) have been intensely studied in the last half of a century. In fact, they were defined by Elie Cartan as early as 1929 with the explicit objective of determining the irreducible components of $\mathbb{L}^{2}(G/K)$ for a large class of Klein pairs $(G,K)$. The following theorem will look like an easy consequence of everything we discussed earlier, but history went the other way and it is in looking for spherical functions that Harish-Chandra discovered what we called Helgason waves.

##### Theorem

(Harish-Chandra 1958, [61])

*In each of the irreducible components*$\mathcal{E}_{\omega}(\mathbb{D})$, *there is a unique spherical function*; *it is the map*

$$\varphi_{\omega}:= x \mapsto\int_{B} e_{\omega,b}(x)\, db. $$

If we plot $\varphi_{\omega}$ it will resemble the Euclidean Bessel-kind covariance functions; only there is a marked difficulty in dealing with the growth at infinity of these functions, which accounts for some (not all, of course) of the many difficulties Harish-Chandra and Helgason had to overcome in developing harmonic analysis on $\mathbb{D}$.

The properties of elementary spherical functions include the conditions which guarantee, thanks to the existence theorem by Kolmogorov mentioned in Sect. 2, that each of the $\varphi_{\omega}$ really is the covariance function of a Gaussian field on $\mathbb{D}$. So we can summarize the preceding discussion with the following statement.

##### Proposition A

*For each*$\omega>0$, *there is exactly one monochromatic Gaussian field on*$\mathbb{D}$*with spectral parameterω*.

These are our candidates for providing V1-like maps on $\mathbb{D}$. We now need to see, by plotting one, whether a “typical” sample of a monochromatic field looks like a hyperbolic V1-like map, hence we need to go from the covariance function to a plot of the field itself. All technical details aside, Euclidean and hyperbolic spherical functions are close enough for the transition from a spherical function to the associated Gaussian field to be exactly the same in both cases. We said nothing of this step in Sect. 2, so let us come back to the Euclidean setting for a second.

Spherical functions there we already described: they are Fourier transforms of the Dirac distribution on a circle, so they read

$$\psi_{R}:= x \mapsto\int_{B} e^{i R b \cdot x}\, db. $$

Now, this builds $\psi_{R}$ out of a constructive interference between plane waves $e_{R,b}$, $b \in B$. In order to obtain a Gaussian random field while keeping the eigenfunction property, we need only attribute a random Gaussian weight (which is a complex number, this includes phase) to each of our plane waves. This needs some care since we are dealing with a continuum of weights to attribute, but there is a standard random measure $\mathbb{Z}$ on the circle, the standard Gaussian white noise, which is meant to achieve this (see the Appendix for details, and also [36]): this produces an invariant random field

$$\mathbf{z}_{R}:= x \mapsto\int_{B} e^{i R b \cdot x} \,d\mathbb{Z}(b) $$

whose covariance is $\psi_{R}$ as desired. We give further details of the transition from $\psi_{R}$ to $\mathbf{z}_{R}$ in the Appendix.

This construction depends only on properties which are common to the Euclidean and hyperbolic plane; thus, it transfers unimpaired to the hyperbolic plane.

We know finally what an orientation preference-like map should look like in hyperbolic geometry: pick a positive number *ω*; out of the stochastic integral

$$x \mapsto\int_{\mathbb {S}^{1}} e_{\omega,b}(x) \,d\mathbb{Z}(b) $$

there will arise orientation maps. Just as in the Euclidean case, they are readily approximated by picking a number of regularly spaced points $b_{1}, \ldots, b_{n}$ on the boundary circle, assigning them independent reduced Gaussian weights $\zeta_{1}, \ldots, \zeta_{n}$ in $\mathbb {C}$ (so the $\zeta_{i}$ are complex-valued reduced Gaussian random variables, independent from each other) and considering

$$x \mapsto\frac{1}{n} \sum_{k=1}^{n} \zeta_{i} e_{\omega, b_{i}}(x). $$

**Fig. 5:**
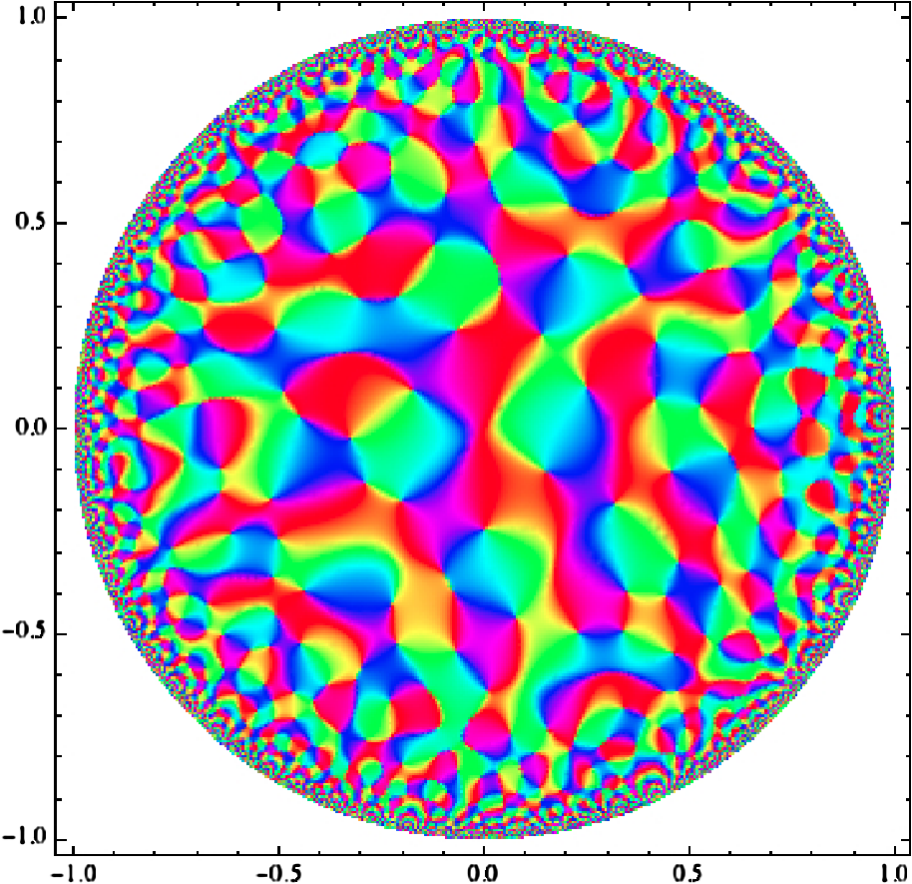
Plot of a monochromatic “orientation map” on the hyperbolic plane. We used the spectral parameter $\omega= 18$ in units of the disk’s radius. Because of the growth factor in the modulus of the $e_{\omega, b}$, drawing a picture in which discretization effects do not appear calls for using more propagation directions than it did in the Euclidean case: 200 directions were used to generate the drawn picture

A computer-generated sample is shown on Fig. 5; comparison with Escher’s celebrated drawings of periodic tilings of $\mathbb{D}$ [64] might be telling.

So there does appear a quasi-periodic tiling of the unit disk; it should not of course be forgotten that this quasi-periodicity holds only when the area of an “elementary cell” is measured in the appropriate hyperbolic units (see the previous section and the next).

#### Hyperbolic Pinwheel Density

What is this area *σ* of an elementary cell, by the way, and can we estimate the density of pinwheels per area *σ*?

In the Euclidean case, we used results from physics that originally dealt with superpositions of Euclidean waves. Of course singularities in superpositions of random waves do occur in many interesting physical problems: interest first came from the study of waves traveling through the (irregular) arctic surface [65]; quantum physics has naturally been providing many interesting random superpositions: they occur in laser optics [66], superfluids [67]… . This has prompted recent mathematical developments. In this section, we would like to point out that these are now sharp enough to allow for calculations *outside* Euclidean geometry.

Consider an invariant monochromatic random field **z**, and write *ω* for the corresponding “wavenumber” (so that **z** belongs to $\mathcal{E}_{\omega}$). We would like to evaluate the expectation for the number of pinwheels (zeroes of **z**) in a given domain $\mathcal {A}$ of the unit disk. Let us write $\mathcal {N}_{\mathcal {A}}$ for the random variable recording the number of pinwheels in $\mathcal {A}$. We will now evaluate the expectation of this random variable, and the result will be summarized as Theorem A below.

Since **z** is *G*-invariant, it is to be expected that $\mathbb {E} \{ \mathcal {N}_{\mathcal {A}} \}$ depends only on the *hyperbolic* area of $\mathcal {A}$: our first claim is that this is indeed the case: writing $|\mathcal {A}|_{h}$ for the hyperbolic area of $\mathcal {A}$, let us show that

$$\frac{\mathbb{E} \{ \mathcal {N}_{\mathcal {A}} \} }{|\mathcal {A}|_{h}} = \frac{V_{0}}{\pi}, $$

where $V_{0}$ stands for the variance of the real-valued random variable $\partial_{x} \mathop{\mathfrak{Re}}(\mathbf{z}) (0)$. For this we shall use Azaïs and Wschebor’s version of the Kac–Rice formula for random fields, which in our setting says the following.

##### Theorem

(see [48], Theorem 6.2)

*Assume***z***is a smooth*, *reduced*^12^*Gaussian random field from*$\mathbb{D}$, *which almost surely has no degenerate zero*^13^*in*$\mathcal {A}$; *then*

$$\mathbb{E} \{ \mathcal {N}_{\mathcal {A}} \} = \frac{1}{2\pi} \int _{\mathcal {A}} \mathbb{E} \bigl\{ \bigl| \det d\mathbf{z}(p) \bigr| | \mathbf{z}(p) = 0 \bigr\} \,dp $$

(*here the integral is Lebesgue integral*, *and the integrand is a conditional expectation*).

To use this theorem, we should note (see [38]) that in a field with constant variance, at each point *p* the value any derivative of any component of the field is independent (as a random variable) from the value of the field at *p*; so the two variables $\operatorname{Det}[d\mathbf {z}](p)$ and $\mathbf{z}(p)$ are independent too; thus for invariant fields on $\mathbb{D}$ we know that the hypotheses in the theorem are satisfied, and that in addition we may remove the conditioning in the expectation formula. So we are left with evaluating the mean determinant of a matrix whose columns are independent Gaussian vectors, with zero mean and the same variance $V_{p}$ as $\partial_{x} \mathop{\mathfrak{Re}}(\mathbf{z}) (p)$. We are left with evaluating the Euclidean area of the random parallelogram generated by these random vectors, and using the “base times height” formula it is easy to prove this mean area is $2V_{p}$.

So we need to see that $\int_{\mathcal {A}} \frac{V_{p}}{\pi}\, dp$ is equal to $|\mathcal {A}|_{h} \frac{V_{0}}{\pi}$. But this is easy: when the real-valued Gaussian field $\zeta= \mathop{\mathfrak{Re}} \mathbf{z} $ is *G*-invariant, we can define a *G*-invariant Riemannian metric on $\mathbb{D}$ by setting $g^{\zeta}_{ij}(p) = \mathbb{E} \{ \partial_{i} \zeta(p) \partial _{j} \zeta(p) \}$; as we said in Sect. 2, this must be a constant multiple of the Poincaré metric. It follows that $V_{p}$ is equal to $\eta(p) V_{0}$, while $\eta(p)$ is the hyperbolic surface element. This proves the announced formula $\mathbb{E} \{ \mathcal {N}_{\mathcal {A}} \} ={|\mathcal {A}|_{h}} \frac{V_{0}}{\pi} $.

Now, evaluating the variance of the first derivative $\partial_{x} \mathop{\mathfrak{Re}}(\mathbf{z}) (0)$ is easy: it is obtained from the *second* derivative with respect to the *x*-coordinate^14^ of the covariance function *Γ* of the random field $\mathop{\mathfrak{Re}}(\mathbf{z})$,

$$\begin{aligned} \mathbb{E} \bigl\{ \bigl(\partial_{1} \mathop{\mathfrak{Re}}(\mathbf{z}) (0) \bigr)^{2} \bigr\} = &\partial_{1,x_{1}}\partial_{1,x_{2}} \mathbb{E} \bigl\{ \mathop{\mathfrak {Re}}(\mathbf{z}) (x_{1})\mathop{\mathfrak{Re}}( \mathbf{z}) (x_{2}) \bigr\} |_{x_{1}=x_{2}=x} \\ =&\partial_{1,x} \partial_{1,y} \varGamma\bigl(\alpha (x)\alpha(y)^{-1}\bigr) |_{x=y=0}, \end{aligned}$$

where *α* is a smooth section of the projection from *G* to $\mathbb{D}$ induced by the action of the origin (such a smooth section does exist). For a *G*-invariant field $\partial_{1,x} \partial_{1,y} \varGamma(\alpha (x)\alpha(y)^{-1}) |_{x=y=0} = \partial_{2,x} \partial_{2,y} \varGamma(\alpha(x)\alpha(y)^{-1}) |_{x=y=0}$; as a consequence $V_{0}$ is half the value of $-\Delta\varGamma$ at zero. Now $\Delta\varGamma = -(\omega^{2} + 1) \varGamma$ and $\varGamma(0) = 1/2$ (the value of the covariance function of all of **z** at zero is one, but here we are dealing only with the real part), so we have obtained the following result.

##### Theorem A

*Suppose***z***is the only complex*-*valued*, *centered Gaussian random field on*$\mathbb{D}$*whose probability distribution is*$\mathit{SU}(1,1)$-*invariant*, *and whose correlation function*, *when turned into a function on*$\mathit{SU}(1,1)$, *is Harish*-*Chandra’s spherical function*$\varphi_{\omega}$. *Consider a Borel subset*$\mathcal {A}$*of*$\mathbb{D}$, *write*$|\mathcal {A}|_{h}$*for its area w*.*r*.*t*. *the Poincaré metric*, *and*$\mathcal {N}_{\mathcal {A}}$*for the random variable recording the number of zeroes of***z***in*$\mathcal {A}$. *Then*

$$\frac{\mathbb{E} \{ \mathcal {N}_{\mathcal {A}} \} }{|\mathcal {A}|_{h}} = \frac{\pi}{\omega^{2} + 1}. $$

It is worth pointing out that the proof above no longer features any reference to wave propagation; we just needed the invariance properties of our covariance function and a nice property of our new Laplacian. This means that calculations should travel unimpaired to geometries where nothing like wave propagation is available for building spherical functions and representation theory. We shall see this at work on the sphere in the next section.

But let us linger a moment in the hyperbolic plane, for our new monochromatic maps do exhibit a rather unexpected feature: while same-phase wavefronts in $e_{\omega,b}$ line up at hyperbolic distance $\frac{2\pi}{|\omega|}$, it seems like the right hyperbolic area for a “hyperbolic hypercolumn”, that area which we called *σ* at the beginning of this subsection, should be $\frac{4\pi^{2}}{\omega^{2} + 1}$. In fact, we claim that the typical hyperbolic distance between two points in the map that have the same orientation preference is *not*$\frac{2\pi}{|\omega|}$ as we would guess by thinking in Euclidean terms, but $\frac{2\pi}{\sqrt{\omega^{2} + 1}}$. There is something of course to support of this claim: we can evaluate the typical spacing by selecting a portion of a geodesic and evaluate the mean number of points with a given orientation preference. To motivate the statement of our result, see the discussion preceding Theorem C below, and also [34].

##### Theorem B

*Suppose***z***is the only complex*-*valued*, *centered Gaussian random field on*$\mathbb{D}$*whose probability distribution is*$\mathit{SU}(1,1)$-*invariant*, *and whose correlation function*, *when turned into a function on*$\mathit{SU}(1,1)$, *is Harish*-*Chandra’s spherical function*$\varphi_{\omega}$. *Select a geodesicδon*$\mathbb{D}$, *consider a segmentΣonδ*, *and write*$|\varSigma|_{h}$*for its hyperbolic length*. *WriteΨfor the real*-*valued random field onΣobtained by projecting the values of*$\mathbf{z}|_{\varSigma}$*onto an arbitrary axis in*$\mathbb {C}$, *and*$\mathcal {N}_{\varSigma}$*for the random variable recording the number of zeroes ofΨonΣ*. *DefineΛas the only positive number such that*$\Delta_{\mathbb{D}} \mathbf{z} = - (\frac{2\pi }{\varLambda} )^{2} \mathbf{z}$. *Then*

$$\frac{\mathbb{E}[\mathcal{N}_{\varSigma}]}{|\varSigma|_{h}} = \frac{1}{\varLambda }. $$

Note that the zeroes considered here are points on *Σ* where the preferred orientation is the vertical, and have nothing to do with pinwheel centers (which were the zeroes considered in Theorem A).

Let us give a summarized proof of this result here (see also the discussion leading to Theorem C, where the arguments are similar but the idea appears perhaps more clearly). Set $\mathbf{u}:= \mathop{\mathfrak {Re}}(\mathbf{z}|_{\delta})$; this is a real-valued random field on the geodesic *δ*. Since *δ* is an orbit on $\mathbb{D}$ of a one-parameter subgroup of $\mathit{SU}(1,1)$, we can view it as a real-valued, stationary random field on the real line and apply the classical one-dimensional Kac–Rice formula. Using the one-parameter subgroup to transfer the result back to *δ*, and using the shift-invariance of **z**, we get the following formula:

$$\frac{\mathbb{E}[ \mathcal{N}_{\varSigma} ]}{|\varSigma|_{h}} = \frac{\sqrt {\lambda_{2}}}{\pi}, $$

where $\lambda_{2} = \mathbb{E}[\mathbf{u}'(0)^{2} ]$ is the second spectral moment of the field **u**. But we actually evaluated $\sqrt{\lambda_{2}}$ while proving Theorem A: it is equal to $\frac{\omega^{2}+1}{\pi}$. This completes the proof of Theorem B.

### Spherical Geometry

Let us now examine the positively-curved case, viz. the sphere $\mathbb {S}^{2}$. Recall from Sect. 2 that the geometry of the sphere is captured by the Klein pair $(\mathit{SO}(3), \mathit{SO}(2))$.

We will start by looking for an orientation preference-like map on the sphere. Let us therefore look for an arrangement **z** with our usual randomness structure, that is, for a complex-valued standard Gaussian random field on the space $\mathbb {S}^{2}$; let us further assume that the field **z** is G-invariant and probes an irreducible factor of the natural representation of $\mathit{SO}(3)$ on $\mathbb{L}^{2}(\mathbb {S}^{2})$ (see Sect. 2.4.2). The arguments we used for the hyperbolic plane go through, so we are now looking for a Gaussian random field whose covariance function is an elementary spherical function for $\mathbb {S}^{2}$.

In the last section, we built these out of hyperbolic analogues of Euclidean plane waves; here there is no obvious “plane wave” candidate for carrying the torch. However, it is quite easy to find alternative building-blocks for the irreducible factors of the representation of *G* on $\mathbb{L}^{2}(\mathbb{S}^{2})$: these are the familiar *spherical harmonics*; since there will be a significant difference between the maps we shall describe and those we encountered on nonpositively curved spaces of the preceding sections, we shall use the next paragraph to examine their rôle in representation theory even if this is famous textbook material; see [68], Chap. 7.

#### Preliminaries on the Spherical Harmonics and the Plancherel Decomposition of $\mathbb{L}^{2}(\mathbb {S}^{2})$

The sphere has its own Laplace operator, just as the Euclidean plane and the hyperbolic plane do. To define it, regard $\mathbb {S}^{2}$ as isometrically embedded in $\mathbb {R}^{3}$ as the unit sphere centered at the origin *O*. If *f* is a smooth function on the sphere, we may extend it to a smooth function $\tilde{f}$ on $\mathbb {R}^{3} -O$ that is constant on every ray issued from the origin: $\tilde{f}(x) = f(x/\|x\|)$. Define now

$$\Delta_{\mathbb {S}^{2}} f = ( \Delta_{\mathbb {R}^{3}} \tilde{f} ) |_{\mathbb {S}^{2}}. $$

Since $\Delta_{\mathbb {R}^{3}}$ is rotation-invariant, $\Delta_{\mathbb {S}^{2}} $ is rotation-invariant also; so every “eigenspace” of $\Delta_{\mathbb {S}^{2}}$ on $\mathbb{L}^{2}(\mathbb {S}^{2})$ is a *G*-invariant subspace. To get eigenfunctions for $\Delta_{\mathbb {S}^{2}}$, we need only remark that if *Y* is a homogeneous function of degree ${\ell+1}$ on $\mathbb {R}^{3}$,

$$(\Delta_{\mathbb {R}^{3}} Y) |_{\mathbb {S}^{2}} = \ell(\ell+1) Y |_{\mathbb {S}^{2}} + \Delta_{s^{2}} ( Y |_{\mathbb {S}^{2}} ) $$

(here *ℓ* is a nonnegative integer).

If we start with a homogeneous function $\varPhi: \mathbb {R}^{3} \rightarrow \mathbb {C}$ of degree ${\ell+1}$ that is in addition *harmonic*, which means that it satisfies $\Delta_{\mathbb {R}^{3}} \varPhi= 0$, and restrict it to the sphere, we get an eigenfunction for $\Delta_{\mathbb {S}^{2}}$, with eigenvalue $\ell(\ell +1)$. Actually, any member of the corresponding eigenspace can be extended to a harmonic homogeneous function of degree ${\ell+1}$, so this describes the whole eigenspace. Now, it turns out that every member of this eigenspace extends to a harmonic homogeneous *polynomial* function of degree $\ell+1$! If we write $\mathcal{H}_{\ell}$ for the $\ell(\ell+1)$-eigenspace of $\Delta_{\mathbb {S}^{2}}$, this space is then finite-dimensional, and its dimension is readily seen to be $2\ell+ 1$. Being finite-dimensional, $\mathcal{H}_{\ell}$ is a closed subspace of $\mathbb{L}^{2}(\mathbb {S}^{2})$, so the usual scalar product on $\mathbb{L}^{2}(\mathbb {S}^{2})$ restricts to a scalar product on $\mathcal{H}_{\ell}$. Here Laplace’s spherical harmonics come into play, for they give an orthonormal basis for $\mathcal{H}_{\ell}$: if we use spherical coordinates $(\theta, \phi)$ on $\mathbb {S}^{2}$ and define, for $\ell \in \mathbb {N}^{\star}$ and $m \in\{-\ell, \ell\}$,

$$Y_{\ell,m}(\theta, \phi):= e^{i m \phi} P_{\ell}(\cos\theta), $$

where $P_{\ell}$ is the *ℓ*th Legendre polynomial, then $\{Y_{\ell , -\ell}, \ldots, Y_{\ell, 0}, \ldots, Y_{\ell, \ell} \}$ is an orthonormal basis for $\mathcal{H}_{\ell}$.

We should add at this point that the natural representation of $\mathit{SO}(3)$ on $\mathcal{H}_{\ell}$ is indeed irreducible; in the next section we shall associate an orientation preference map to each of the $\mathcal {H}_{\ell}$. But before we close this section, let us see how this relates to the decomposition of $\mathbb{L}^{2}(\mathbb {S}^{2})$.

Starting from an element $f \in\mathbb{L}^{2}(\mathbb {S}^{2})$, we may produce a countable set of coefficients by setting, for each $\ell\in \mathbb {N}^{\star}$ and each $m \in\{-\ell, \ldots, \ell\}$,

$$\widehat{f}(\ell, m):= \int_{\mathbb {S}^{2}} f(x) Y_{\ell, m}(x) \,dx. $$

For each value of *ℓ*, this yields an eigenfunction of Δ related to *f*, namely $f_{\ell}:= x \mapsto\sum_{m} \widehat{f}(\ell , m) Y_{\ell, m}(x)$. We are thus defining a *projection operator*$\mathcal{P}_{\ell}: \mathbb{L}^{2}(\mathbb {S}^{2}) \rightarrow\mathcal {H}_{\ell}$ (note that $\mathcal{P}_{\ell}^{2} = \mathcal{P}_{\ell}$).

Now, it is a very famous theorem of Hermann Weyl that the initial map *f* can be reconstructed from this generalized Fourier series:

$$f = \sum_{\ell\geq0} f_{\ell}. $$

Here, convergence of the right-hand side is to be understood in the mean-quadratic sense; but if *f* is smooth, uniform convergence does hold.

Notice that if *f* were to be an eigenfunction of $\Delta_{\mathbb {S}^{2}}$ but were to belong to none of the $\mathcal{H}_{\ell}$, $\mathcal{P}_{\ell } f$ would be zero for every *ℓ*, and so would *f*: Weyl’s theorem thus indicates that there is no other eigenvalue of $\Delta_{\mathbb {S}^{2}}$ (thus the Peter–Weyl theorem reduces to the spectral theorem for the hermitian operator $\Delta_{\mathbb {S}^{2}}$ in the special case considered here). Of course it also achieves the Plancherel decomposition of Sect. 2.4.2,

$$\mathbb{L}^{2}\bigl(\mathbb {S}^{2}\bigr) = \bigoplus _{\ell\geq0} \mathcal{H}_{\ell }. $$

Notice that all analytic difficulties in the decomposition have vanished (the irreducible factors $\mathcal{H}_{\ell}$ are really spaces of square-integrable functions), and that Fourier *series* are enough to reconstruct a function, which means here that a countable set of irreducible representations is enough to decompose $\mathbb {L}^{2}(\mathbb {S}^{2})$. Recall from Sect. 2.4.2. that there is a simple reason for the marked differences between what happens on the sphere and what happens in our previous examples: Hermann Weyl proved that when the group *G* is *compact*, there is but a countable set of equivalence classes of irreducible representations.

#### Spherical Orientation Maps

We have now at our disposal everything that is needed for orientation preference-like maps on the sphere, and on top of it, one important observation: our set of spherical maps, unlike the set of its Euclidean or Hyperbolic analogues, is discrete in nature. Out of the spherical harmonics $Y_{\ell m}$ arises one irreducible factor of $\mathbb{L}^{2}(\mathbb {S}^{2})$ per *ℓ*; we feel it is appropriate to name the corresponding invariant Gaussian random field a *spinℓmonochromatic field*.

In the Euclidean and hyperbolic cases, we got all the information from the covariance function of the field; here we can dispense with the covariance function and describe such a field, say $\varPhi_{\ell}$, a bit more explicitly than we could do for the previous invariance structures. Since the representation space $\mathcal{H}_{\ell}$ is finite-dimensional, specifying an orthonormal basis $(Y_{\ell,m})_{m}$ for $\mathcal{H}_{\ell}$ easily yields a Gaussian probability law on $\mathcal{H}_{\ell}$: we need only consider

$$\sum_{m = -\ell}^{\ell} \zeta_{m} Y_{\ell, m}, $$

where the $\zeta_{m}$ are reduced complex-valued Gaussian random variables independent from each other. Now $\mathcal{H}_{\ell}$ may be seen as a function space on $\mathbb{S}^{2}$, with the corresponding functions readily written

$$\varPhi_{\ell}: x \mapsto\sum_{m = -\ell}^{\ell} \zeta_{m} Y_{\ell, m} (x). $$

Thus, a standard Gaussian probability law on $\mathcal{H}_{\ell}$ defines a Gaussian random field. Now *G* acts on $\mathcal{H}_{\ell}$ by unitary operators, and the probability density $\sum\zeta_{m} Y_{\ell, m}$ is *G*-invariant; this means that

$$x \mapsto\sum_{m = -\ell}^{\ell} \zeta_{m} Y_{\ell, m} (x) $$

is an invariant Gaussian field that spans the “spin *ℓ*”-irreducible subspace of $\mathbb{L}^{2}(\mathbb {S}^{2})$. This is precisely our spin *ℓ* monochromatic field; it is the only *G*-invariant standard Gaussian field with values in $\mathcal{H}_{\ell}$.

**Fig. 6:**
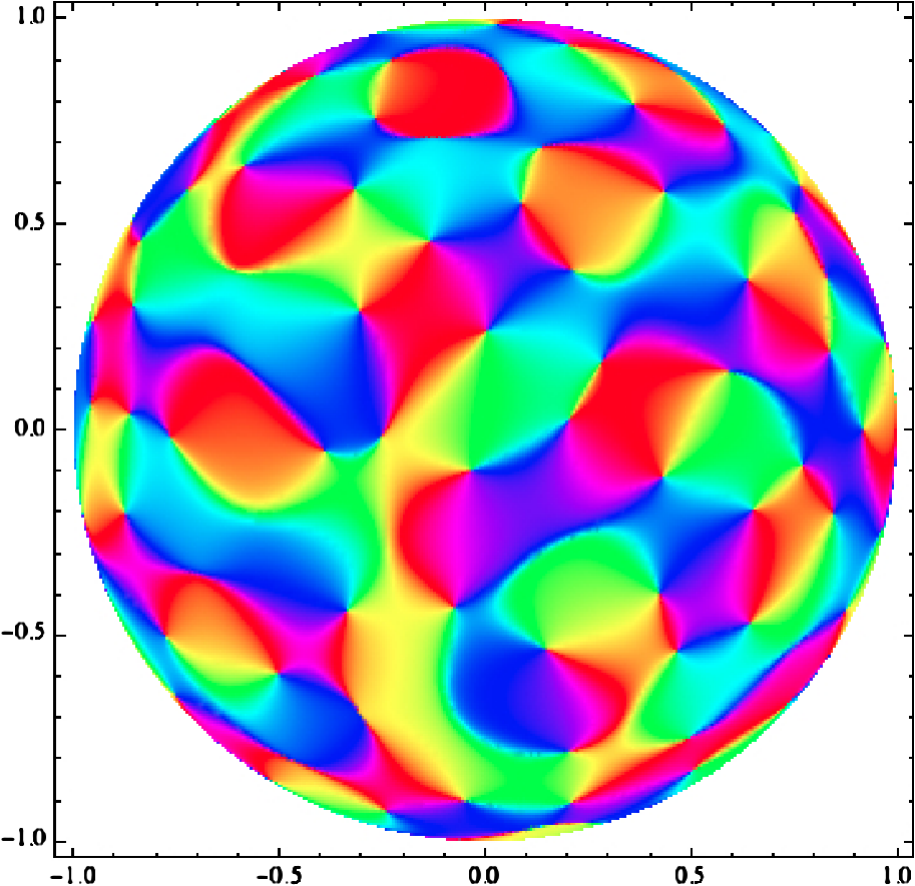
An orientation map on the sphere sampled from the argument of a monochromatic $\mathit{SO}(3)$-invariant Gaussian random field on the sphere with spin 7. We plotted the restriction to a hemisphere; we used a superposition of spherical harmonics with spin seven and random, reduced independent Gaussian weights

We have plotted a map sampled from this field on Fig. 6.

#### Spherical Pinwheel Density

Expectation values for pinwheel densities in spherical maps may be evaluated with the same methods we used in the previous sections. Here, however, there appears a significant difference with the Euclidean and Hyperbolic cases: while monochromatic fields in those cases were indexed by a continuous parameter that is easily interpreted as a wavelength, there is apparently no natural scale for writing pinwheel densities.

In this subsection, we shall answer the following two questions:

- What is the mean (spherical) distance *Λ* between iso-orientation domains in a field that probes $\mathcal{H}_{\ell}$?
- What is the mean number of pinwheels within a given subset of the sphere, relative to the (spherical) area of this subset? Is it $\frac {\pi}{\varLambda^{2}}$?

To answer the first question, let us select a geodesic segment on $\mathbb {S}^{2}$, that is, a portion of a great circle. What is, on this segment, the mean number of points where $\varPhi_{\ell}$ exhibits a given orientation? Since standard Gaussian fields are shift-invariant, we can consider a fixed value of the orientation, say the vertical. Points where $\varPhi_{\ell}$ exhibits this orientation are points where $\mathop{\mathfrak{Re}}(\varPhi_{\ell})$ vanishes; so let us define $\varPsi_{\ell} = \mathop{\mathfrak{Re}}(\varPhi_{\ell})$ and look for its zeroes on the given great circle.

Now, $\varPsi_{\ell}$ is a Gaussian field on our great circle that is invariant under any rotation around this circle. This may be thought of as a *stationary* (translation-invariant) random field on $\mathbb {R}$—an instance where the classical Kac–Rice formula [38] applies (think of what happens if one rolls this circle around on a Euclidean plane at constant speed). So we may assert that if $\mathcal {N}_{\varSigma}$ is the random variable recording the number of zeroes of $\varPsi_{\ell}$ on *Σ*,

$$\frac{\mathbb{E}[ \mathcal{N}_{\varSigma} ]}{|\varSigma|} = \frac{\sqrt {\lambda_{2}}}{\pi}, $$

where $\lambda_{2} = \mathbb{E}[ \varPsi_{\ell}''(0)^{2} ]$ is the second spectral moment of the field $\varPsi_{\ell}$. But now $\lambda_{2} = \frac{\ell(\ell+1)}{4}$; if we set

$$\varLambda:= \frac{2\pi}{\sqrt{\ell(\ell+1)}} $$

we have then obtained the following result.

##### Theorem C

*suppose*$\varPhi_{\ell}$*is the only complex*-*valued*, *centered Gaussian random field on*$\mathbb{S}^{2}$*whose probability distribution is rotation*-*invariant*, *and whose samples belong to the irreducible subspace of*$\mathbb{L}^{2}(\mathbb{S}^{2})$*spanned by the spherical harmonics*$Y_{\ell m}$, $m = -\ell, \ldots, \ell $. *Consider a geodesic segmentΣon*$\mathbb{S}^{2}$, *write*$\mathcal{N}_{\varSigma}$*for the random variable recording the number of points onΣwhere*$\varPhi_{\ell}$*takes real values*, *and writeΛfor the positive number*$\frac{2\pi}{\sqrt{\ell(\ell+1)}}$. *Then*

$$\frac{\mathbb{E}[ \mathcal{N}_{\varSigma} ]}{|\varSigma|} = \frac {1}{\varLambda}. $$

Thus, if the mean number of points on *Σ* to which $\varPhi_{\ell}$ attributes a given orientation preference is to be no less or no more than one, the length of *Σ* must be *Λ*. This answers question (a).

Now, if $\mathcal{A}$ is a subset of the sphere, denote by $|A|_{s}$ its spherical area and by $\mathcal{N}_{\mathcal{A}}$ the random variable recording the number of pinwheels of $\varPhi_{\ell}$ in *A*. Then, as in the previous cases, we observe a scaled density of *π*:

##### Theorem D

*Suppose*$\varPhi_{\ell}$*is the only complex*-*valued*, *centered Gaussian random field on*$\mathbb{S}^{2}$*whose probability distribution is rotation*-*invariant*, *and whose samples belong to the irreducible subspace of*$\mathbb{L}^{2}(\mathbb{S}^{2})$*spanned by the spherical harmonics*$Y_{\ell m}$, $m = -\ell, \ldots, \ell $. *Consider a Borel subset*$\mathcal {A}$*of*$\mathbb{S}^{2}$, *write*$|\mathcal {A}|_{s}$*for its area w*.*r*.*t*. *the round metric*, *and*$\mathcal {N}_{\mathcal {A}}$*for the random variable recording the number of zeroes of***z***in*$\mathcal {A}$. *Set*$\varLambda= \frac{2\pi}{\sqrt {\ell(\ell+1)}}$*as above*. *Then*

$$\frac{\mathbb{E} \{ \mathcal {N}_{\mathcal {A}} \} }{|\mathcal {A}|_{s}} = \frac{\pi}{\varLambda^{2}}. $$

Let us give a sketch of proof of Theorem D: since the only difference with the hyperbolic case is the lack of global coordinates which simplified the presentation there, we think it is better to keep this proof short and refer to our upcoming Ph.D. thesis for full details. A first step is to adapt the formula by Azais and Wschebor (the version in Sect. 3.1.4 holds when the field is defined on an open subset of $\mathbb {R}^{n}$) to prove that $\mathbb{E} \{ \mathcal {N}_{\mathcal {A}} \} ={|\mathcal {A}|_{s}} \frac{V_{0}}{\pi}$, where $V_{0}$ is the variance of any derivative of $\mathop{\mathfrak{Re}}(\mathbf{z})$ at a point $p_{0}$ on $\mathbb{S}^{2}$. Now to evaluate $V_{0}$, we use the fact that it is equal to the expectation for the second partial derivative (in any direction) at $p_{0}$ of the covariance function *Γ* of $\mathop{\mathfrak{Re}}(\mathbf{z})$. This expectation does not depend on the chosen direction, and to adapt the arguments in the proof of Theorem A we can use the group-theoretical interpretation of $\Delta_{\mathbb {S}^{2}}$ as the Casimir operator associated to the action of $\mathit{SO}(3)$ on $\mathbb{S}^{2}$ (see [43], Sect. 5.7.7). As in the proof of Theorem A, we can then evaluate $V_{0}$ as half the value of $\Delta_{\mathbb{S}^{2}}(\varGamma)$ at $p_{0}$, but because $\Delta_{\mathbb {S}^{2}} \mathbf{z} = (\frac{2\pi}{\varLambda} )^{2} \mathbf{z}$ this half-value turns out to be $\frac{\pi}{\varLambda}^{2}$, proving Theorem D.

#### An Alternative Orientation Map, with Shift-Twist Symmetry

We have so far been looking for arrangements of V1-like receptive profiles on curved (homogeneous) surfaces; for this we used complex-valued random fields. We shall now look for a pinwheel-like structure on the sphere which is of a slightly different kind, perhaps more likely to be of use in discussions which include horizontal connectivity, or which relate to the vestibular system and its interaction with vision. We will also provide a simple criterion on pinwheel densities to distinguish between our two types of spherical maps.

In this subsection $\mathbb {S}^{2}$ sits as the unit sphere in $\mathbb {R}^{3}$, and we try to arrange *three*-dimensional abelian Fourier coefficients on the sphere: in other words, we assume each point $\vec{u}$ on $\mathbb {S}^{2}$ is equipped with a sensor whose receptive profile depends on a plane wave $x \in \mathbb {R}^{3} \mapsto \operatorname{exp} \langle\omega(\vec{u}) \cdot x\rangle$ (this profile could be a three-dimensional Gabor wavelet). Here $\omega(\vec{u}) \in \mathbb {R}^{3}$ is a linear form on $\mathbb {R}^{3}$ (so it may be thought of as a vector). Let us assume further that at each point $\vec{u}$, the corresponding sensor neglects everything that happens in directions collinear to $\vec{u}$, so that $\omega(\vec{u})\cdot v = 0$ as soon as $\vec{v} \perp\vec{u}$.

This kind of arrangement does not seem very interesting if (a part of) the sphere is thought of as a piece of cortical surface, and we do not set it forth as a model for a visual area; yet it would not be completely unreasonable to think of an arrangement like this if $\vec {u}$ were to stand for gaze direction, and it makes sense (not to say that it is useful) to consider a remapping of this structure across the cortical surface (this would displace the interpretation of the pinwheel-like layout, which would only exist at a functional level).

Now, there is a natural operation of the rotation group $\mathit{SO}(3)$ on such arrangements: if *R* is a rotation and *ω* is a map as above, then the natural “rotated *ω*”, viz.

$$\vec{u} \overset{R_{\star} \omega}{\longmapsto} R \cdot\omega \bigl(R^{-1} \vec{u}\bigr) $$

is an arrangement of the same kind. Notice that if *R* is a rotation of axis $\vec{u}$, it shifts the “orientation preference” in $\omega(\vec{u})$.

This formula is familiar from differential geometry; in fact, our set of maps is precisely the set $\varOmega^{1}(\mathbb {S}^{2})$ of (vector fields or, more accurately) *differential 1-forms* on the sphere. Now, let us come back to $\varOmega^{1}(\mathbb {S}^{2})$: we can add two such maps, so $\varOmega ^{1}(\mathbb {S}^{2})$ is a vector space. After a suitable completion, we may consider the Hilbert space $\varOmega^{1}_{\mathbb{L}^{2}}(\mathbb {S}^{2})$ of forms which are square-integrable, and since rotations are unitary maps, and writing $P(g)$ for the map $\omega\mapsto g_{\star} \omega$ whenever *g* is a rotation, we get a unitary representation $(\varOmega^{1}_{\mathbb {L}^{2}}(\mathbb {S}^{2}), P)$ of the rotation group.

Using this representation may look rather unnatural in biology; but corresponding transformations *have* been discussed in the flat case, though with a very different language: in [27, 31] they are called *Shift-twist transformations*. Indeed, differential forms on $\mathbb {R}^{2}$ can be identified with functions from $\mathbb {R}^{2}$ to $\mathbb {C}$, and the natural action on differential forms of a rotation around the origin^15^$(A, 0) \in \mathit{SE}(2)$ is turned in this way into the operation $f \mapsto A f( A^{-1} \cdot)$ on complex-valued functions, which is exactly the shift-twist transformation considered in [27, 31] (compare Sect. 2.3 in [27]).

Bringing the horizontal connectivity and notions like the association field into the picture ([13, 30], Chap. 4), it seems natural to introduce the (co-)tangent bundle of the surface on which orientation maps are to be developed.

Now, the unitary representation $(\varOmega^{1}_{\mathbb{L}^{2}}(\mathbb {S}^{2}), P)$ is of course not irreducible; so in order to get “elementary arrangements”, we may look for its irreducible constituents as we did for $\mathbb{L}^{2}(\mathbb {S}^{2})$ and hope that pinwheel-like structures are to be found there.

There is a useful remark here: if *f* is a real-valued smooth function on the sphere, its derivative *df* provides us with an element of $\varOmega^{1}_{\mathbb{L}^{2}}(\mathbb {S}^{2})$. What is more, if *g* is a rotation, then $P(g) \,df = d [ \vec{u} \mapsto f(g^{-1} \vec{u}) ]$. So any *G*-invariant irreducible subspace $\mathcal{H}_{\ell}$ of $\mathbb {L}^{2}(\mathbb {S}^{2})$ yields a *G*-invariant irreducible subspace $\mathcal{H}_{\ell}^{\mathrm{exact}}$ of $\varOmega^{1}_{\mathbb{L}^{2}}(\mathbb {S}^{2})$: we need only consider the derivatives of real parts of elements of $\mathcal{H}_{\ell}$.

**Fig. 7:**
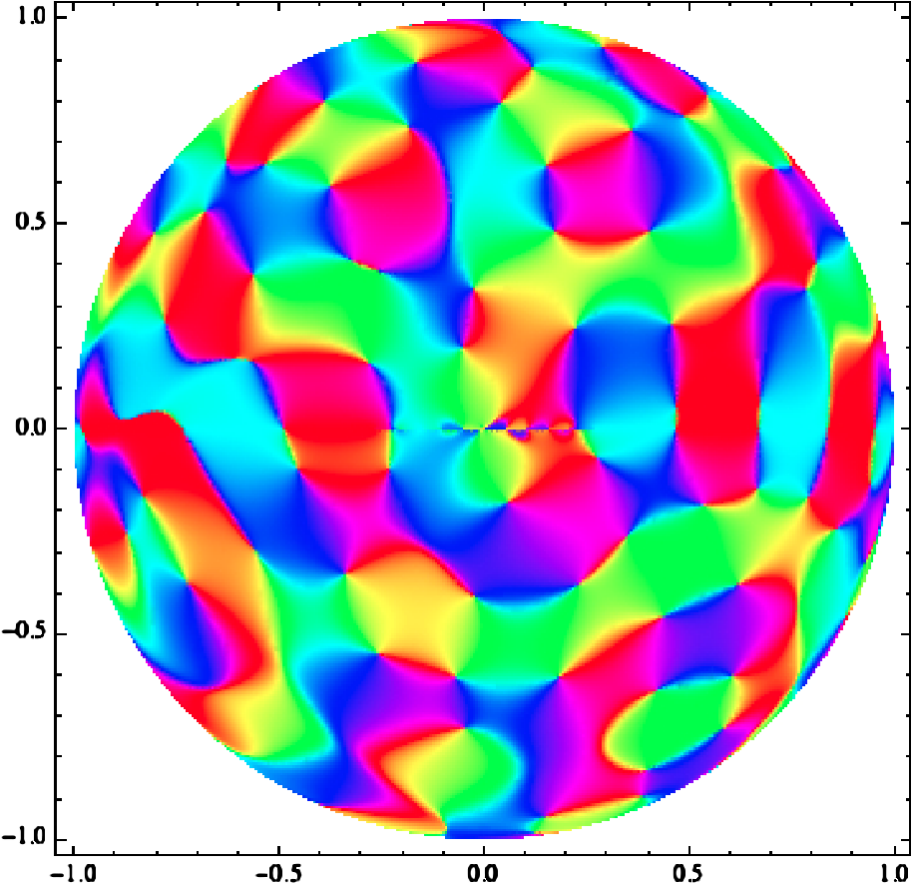
An orientation map on the sphere sampled from a random vector field which has $\mathit{SO}(3)$-shift-twist symmetry. We plotted the restriction to a hemisphere of the random map exploring $\mathcal{H}^{\mathrm{exact}}_{10}$; beware that the color coding has a different meaning than in Figs. 1, 2, 5, and 6. Here, the sample map is a vector field on the sphere, and there is no complex number; to visualize the direction of the emerging vector at each point, we apply the orthogonal projection from the drawn hemisphere to the “paper” plane, thus getting a vector field on the unit ball of the Euclidean plane, and plot the resulting orientation map using the same color code as in Figs. 1, 2, 5, and 6

All in all, if we start with one of the monochromatic random fields $\varPhi_{\ell}$, $\ell\geq1$ and consider the derivative of its real part, we get a random differential form $\varpi_{\ell}$ on $\mathbb {S}^{2}$ which probes one irreducible factor of $\varOmega^{1}(\mathbb {S}^{2})$. What kind of “orientation map” does this correspond to? Plotting this needs a warning: when *ω* is a differential form the $\omega(\vec{u})$ appear in different tangent planes as $\vec{u}$ varies, so a picture may be misleading; luckily it is orientation maps we wish to plot, and the projections of the $\omega(\vec{u})$ on a plane through zero give a fine idea of the layout of orientations on each of the hemispheres it cleaves $\mathbb {S}^{2}$ into. A plot of a projection of $\varpi_{\ell}^{\mathrm{exact}}$ for $\ell= 10$ is displayed on Fig. 7.

Is there more to fields probing other irreducible factors of $\varOmega^{1}_{\mathbb{L}^{2}}(\mathbb {S}^{2})$ than what we see on the $\mathcal{H}_{\ell}^{\mathrm{exact}}$? There is not, for there is a duality operation on $\varOmega^{1}_{\mathbb{L}^{2}}(\mathbb {S}^{2})$ which will allow us to describe all the other irreducible factors. This is the Hodge star: to define it in our very particular case, notice first that if $\vec{u}$ is a unit vector, we get an notion of oriented bases on the plane $\vec{u}^{\perp}$ from “the” usual notion of oriented basis in the ambient space. Then, start with a differential form *ω*, and shift each of the (co)-vectors $\omega(\vec{u})$ with a rotation of angle $+\frac{\pi}{2}$ in each (co) tangent space; this gives a new form ✩*ω*. Obviously it is orthogonal to *ω*, and what is more, it commutes with rotations: $g_{⋆}(\text{✩}\omega )=\text{✩}(g_{⋆}\omega )$ for any rotation *g*.

Let us write $\mathcal{H}_{\ell}^{\mathrm{coexact}}$ for the image of $\mathcal {H}_{\ell}^{\mathrm{exact}}$ under the Hodge star; since the Hodge star is a *G*-invariant isometry of $\varOmega^{1}_{\mathbb{L}^{2}}(\mathbb {S}^{2})$, it is a *G*-invariant irreducible subspace too. Now, using a fundamental result of differential geometry (the Hodge–de Rham theorem), we can deduce from this the decomposition

$$\varOmega^{1}_{\mathbb{L}^{2}}\bigl(\mathbb {S}^{2}\bigr) = \bigoplus _{\ell> 0} \bigl( \mathcal{H}_{\ell}^{\mathrm{exact}} \oplus\mathcal{H}_{\ell}^{\mathrm{coexact}} \bigr), $$

where the direct sum is orthogonal.

Random differential forms probing the $\mathcal{H}_{\ell}^{\mathrm{coexact}}$ have exactly the same orientation preference layout as those we have already met, except for a difference of “chirality” that corresponds to a global shift of the orientations. We should note here that (the probability distributions for) our nontwisted fields $\varPhi_{\ell}$ were unchanged under a global shift of the orientations.

While our new maps do resemble the nontwisted orientation maps of the previous paragraph, looking at pinwheel densities will reveal a notable difference. Indeed, although there is a formula of Kac–Rice type for the mean number of critical points of an invariant monochromatic field like $\mathop{\mathfrak{Re}}(\varPhi_{\ell})$, it involves a Hessian determinant at the place where we earlier met the Jacobian determinant of $\varPhi_{\ell}$—this was the determinant of a random matrix with independent coefficients, which is not the case for any Hessian (symmetric!) matrix.

We now need to deal with the mean determinant of a random matrix whose coefficients have Gaussian distributions but exhibit nontrivial correlations. This seems intractable in full generality; fortunately, our specific spherical problem has been solved recently: in ref. [69], the author proves that the mean total number of critical points of a monochromatic Gaussian invariant field, that is, the expectation for the total number of pinwheels (beware this is not a density) in $\varpi_{\ell}^{\mathrm{exact}}$, is equivalent to

$$\frac{\ell^{2}}{\sqrt{3}} $$

as *ℓ* goes to infinity. Actually, for “finite” *ℓ*, the total number is given by an explicit but complicated expression.

Note that in our nontwisted, complex-valued random fields $\varPhi_{\ell }$, the expectation for the total number of pinwheels is equivalent to

$$\ell^{2} $$

as *ℓ* goes to infinity. So it is easy, at least in principle, to distinguish the two kinds of orientation maps: one needs only a single quantitative measurement.

## Discussion

In this paper, we started from a reformulation of existing work by Wolf, Geisel and colleagues, with the aim to understand the crucial symmetry arguments used in models with the help of noncommutative harmonic analysis, which is often a very well-suited tool for using symmetry arguments in analysis and probability. Understanding these Euclidean symmetry arguments from a conceptual standpoint showed us that Euclidean geometry at the cortical level is a way to enforce conditions that are not specific to Euclidean geometry but have a meaning on every “symmetric enough” space, and we thus saw how a unique Gaussian random field providing V1-like maps can be associated to each irreducible “factor” in the Plancherel decomposition of the Hilbert space of square-integrable functions on the Euclidean plane, the hyperbolic plane and the sphere. We proved that in these three settings, when scaled with the typical value of column spacing, monochromatic invariant fields exhibit a pinwheel density of *π*. Theorems A′ and D′ in the Appendix prove that the same result holds when the monochromaticity condition is dropped: in other words, a pinwheel density of *π* appears as a signature of (shift) symmetry. Since pinwheel densities can be measured in individual sample maps thanks to the ergodicity properties of invariant Gaussian fields (see [38], Sect. 6.5), this yields a criterion to see whether an individual map (which cannot be itself invariant!) is likely to be a sample from a field with an invariance property, whether the map be drawn on a flat region or on a curved, homogeneous enough region. In the spherical case, also we saw that the number of pinwheels in the map can in principle distinguish between rotation-invariance and shift-twist symmetry; to see whether this observation can be turned into a precise criterion distinguishing the various kinds of invariance from actual measurements on individual sample maps, it would probably be interesting to see whether there is anything to be said of pinwheel densities in Euclidean or hyperbolic maps with shift-twist symmetry, and of the mean column spacing in shift-twist symmetric maps.

Since our aim was to understand the role of symmetry arguments, one aspect restricting the scope of our constructions in a fundamental way is our focusing on homogeneous spaces rather than spaces with variable curvature. Of course, we have good technical reasons for this: the way symmetry arguments are used in existing discussions made it natural to focus on those two-dimensional spaces which have a large enough symmetry group, and our constructions are entirely based on exploiting the presence of this symmetry group. One might wish to make the setting less restrictive, especially since the places where the surface of real brains is closest to a homogeneous space are likely to be the flat parts. But using analogues of symmetry arguments on nonsymmetric spaces is a major challenge in (quantum) field theory, and if one wished to start from the reformulation we gave of Wolf and Geisel’s work in Sect. 2.4, generalizing the arguments of this paper to find V1-like maps on Riemannian manifolds on nonconstant curvature would be formally analogous to adapting Wigner’s description of elementary particles on Minkowski spacetime to a general curved spacetime—a challenge indeed! Answering this challenge would bring us close to the two-dimensional models from quantum field theory or statistical mechanics, and make us jump to infinite-dimensional “phase spaces” (and would-be groups). This is a step the author is not ready to take, and it is likely that simpler ways to study the nonhomogeneous case would come with shifting the focus from mature maps back to development models.

Indeed, readers familiar with development models have perhaps been puzzled by another aspect of our paper, which is the fact that we used Gaussian random fields as the setting for our constructions: Gaussian fields provide sample maps which look very much like orientation maps, and as we emphasized the statistical properties of their zero set are very strikingly reminiscent of what is to be found in real maps, but there are appreciable and measurable differences between the output of invariant Gaussian fields and real orientation maps (see for instance a discussion in [34]). As we recalled in the Introduction, it is likely that Gaussian fields provide a better description for the early stage of cortical map development, but that the Gaussian description later acquires drawbacks because it is not compatible with the nonlinearities essential to realistic development scenarii.

To our knowledge, many of the most successful models for describing the mature stage of orientation preference maps are variations on the long-range interaction model of Wolf, Kaschube et al. [11, 70, 71], the mature map **z** evolves from an undetermined (random) initial stage (not assumed to be Gaussian) through

$$\partial_{t} \mathbf{z} = L_{\varLambda} (\mathbf{z}) + N_{\gamma, \sigma} (\mathbf{z}). $$

Here $L_{\varLambda}$ is a Swift–Hohenberg operator

$$\mathbf{z} \mapsto r \mathbf{z} -\biggl(\biggl(\frac{2\pi}{\varLambda} \biggr)^{2} + \nabla ^{2}\biggr)^{2} \mathbf{z}, $$

*γ* is a real number between zero and two, *σ* is a positive number and $N_{\gamma, \sigma}$ is the following nonlinear operator:

$$\begin{aligned} N[\mathbf{z}]:= x \mapsto&(1 -\gamma) \bigl|\mathbf{z}(x)\bigr|^{2} \mathbf{z}(x) \\ &{}-(2-\gamma) \int_{\mathbb {R}^{2}} K_{\sigma}(x -y) \biggl( \mathbf{z}(x) \bigl|\mathbf {z}(y)\bigr|^{2} + \frac{1}{2} \bar{ \mathbf{z}}(x) \mathbf{z}(y)^{2} \biggr) \,dy. \end{aligned}$$

Allowing **z** to evolve from an initial fluctuation, when $\gamma< 1$ and when $\sigma/\varLambda$ is large enough Eq. (1) leads first to an invariant, approximately Gaussian field (thanks to an application of the central limit theorem to a linearized version of Eq. (1); see [26]), then to non-Gaussian quasiperiodic V1-like random fields.

This interaction model can easily be adapted to define a nonlinear partial differential equation on any Riemannian manifold: a Riemannian metric, say on *M*, comes with a natural Laplacian $\Delta_{M}$ and a volume form $d\mathit{Vol}_{M}$, so we can define $L^{M}_{\varLambda}$ as

$$\mathbf{z} \mapsto r \mathbf{z} -\biggl(\biggl(\frac{2\pi}{\varLambda} \biggr)^{2} + \Delta_{M}\biggr)^{2} \mathbf{z}, $$

and use the geodesic distance $d_{M}(x,y)$ between any two points *x*, *y* of *M* to define

$$\begin{aligned} N^{M}_{\gamma}[\mathbf{z}]:= x \mapsto&(1 -\gamma) \bigl| \mathbf{z}(x)\bigr|^{2} \mathbf{z}(x) \\ &{}-(2-\gamma) \int_{M} e^{- \frac{d_{M} (x,y)^{2}}{2\sigma^{2}}} \biggl( \mathbf{z}(x) \bigl|\mathbf{z}(y)\bigr|^{2} + \frac {1}{2} \bar{\mathbf{z}}(x) \mathbf{z}(y)^{2} \biggr) \,d \mathit{Vol}_{M}(y). \end{aligned}$$

A non-Euclidean version of the long-range interaction model on *M* would then simply be

1 $$ \partial_{t} \mathbf{z} = L^{M}_{\varLambda} (\mathbf{z}) + N^{M}_{\gamma, \sigma} (\mathbf{z}) . $$

As Wolf and coworkers point out (see for instance the supplementary material in [11], Sect. 2), this partial differential equation is the Euler–Lagrange equation of a variational problem, so solutions are guaranteed to converge to stable stationary states as time wears on. Wolf and colleagues showed (using numerical studies) that when $\sigma/\varLambda$ is large enough, V1-like maps are among the stable solutions in the Euclidean case. On arbitrary Riemannian manifolds, however, there is no way to guarantee that structure-rich stable solutions of the above PDE exist; it would certainly be worth examining, at least with numerical simulations, but this is beyond the author’s strengths at present. It is perhaps natural to imagine that given the analogy between maps obtained by truncation from invariant GRFs and the output of the long-range interaction model, the constructions in this paper are a strong indication that on symmetric spaces, the stable solutions of (1) include maps which look like those of Figs. 5–7, and that the difference between those and the monochromatic invariant fields studied in this paper is analogous to the difference between experimental maps, or at least the output of (1) in the Euclidean case, and maps sampled from invariant Gaussian fields on $\mathbb {R}^{2}$.

In adult animals measurements seem to indicate that the structure of mature maps departs from that of maps sampled from invariant Gaussian fields; remarkably, there is experimental evidence for the fact that a pinwheel density of *π*, which in a Gaussian initial stage appears as a signature of Euclidean symmetry as we saw, is maintained during development in spite of the important refinements in cortical circuitry and the departure from Gaussianity that they induce [11]. Independently of modeling details, we see that geometrical invariance can be measured in principle, even on individual maps: upon evaluating local column spacings (with respect to geodesic length in the curved case) and performing space averaging, one gets a length scale *Λ*; when scaling pinwheel density with respect to *Λ*, observing a value of *π* is a strong indication that geometrical invariance on the cortical surface is an important ingredient in development.

In addition to this, one might think that arranging neurons and their receptive profiles on a homogeneous enough space has benefits from the point of view of information processing: by allowing the conditions of homogeneity and isotropy to make sense, a constant curvature could help distribute the information about the stimulus in a more uniform way (note that as the eyes move constantly, a given image is processed by many different areas in V1 in a relatively short time). Neurons receiving inputs from several adjacent regions of V1 could then handle spike statistics which vary little as the sensors move and have a more stable worldview.

## Appendix A: Riemannian Metrics on Homogeneous Spaces

We tried to make the mathematical prolegomena to the main text lighter by delaying to this Appendix a simple (and well-known) detail on the way to recover the usual metrics on the sphere and the hyperbolic plane from the relevant groups. We will rephrase in a general setting what we said for the hyperbolic plane, and it will be enough to fill the gap.

• Let us start with a group *G* and a compact subgroup *K*, and consider the homogeneous space $X = G/K$. If we are given a scalar product $\langle\cdot, \cdot\rangle_{o}$ on the tangent space $T_{\{K\} } X$ to *X* at the identity coset $o = \{K\}$, and if the action of *K* on this tangent space is isometric for this scalar product, we can define a scalar product at each point *p* of *X*: since there is an element *g* of *G* which takes *p* to *o*, when we consider two vectors $\vec{X}$ and $\vec{Y}$ in $T_{p} X$ we can look at

$$\langle\vec{X}, \vec{Y} \rangle_{p}:= \bigl\langle dg(p) \cdot \vec{X}, dg(p) \cdot\vec{Y} \bigr\rangle _{o}. $$

Then $\langle\vec{X}, \vec{Y} \rangle_{p}$ does not depend on the choice of the element *G*; we have thus defined a Riemannian metric on $X = G/K$, and of course it is *G*-invariant.

• Now there are many important examples of pairs $(G,K)$ for which, up to multiplication by a positive constant, there is only one *K*-invariant scalar product on $T_{\{K\}} X$: this is the case in the two situations considered in our manuscript (in both cases, *K* is the rotation group of an Euclidean plane with a chosen origin, and there is only one rotation-invariant scalar product on the plane).

• Since the usual round metric on $\mathbb{S}^{2}$ is obviously rotation-invariant, what precedes justifies our claim that it can be recovered from group theory (and the global scale factor amounts to choosing a radius for the sphere when we want to embed it in $\mathbb {R}^{3}$ in the usual way).

• In the hyperbolic case, a simple calculation is enough to prove that invariant metrics on $\mathbb{D}$ are constant multiples of the Poincaré metric. It is then simple, as we did in the main text, to describe all geodesics of $\mathbb{D}$ from a single one among them (see ref. [53], p. 29).

## Appendix B: Sampling from Monochromatic Fields in the Euclidean and Hyperbolic Planes

In the main text, we defined monochromatic invariant Gaussian random fields on homogeneous spaces through their correlation functions, relying on an existence theorem by Kolmogorov to pass from the correlation function to the actual field. On Figs. 2 and 6 we display maps sampled from monochromatic invariant Gaussian fields, but we did not say precisely how the drawn object was built from its correlation function. We provide some details in this section.

Let us focus on the Euclidean case to simplify notation; as we saw in Sect. 3.2.2, this actually covers the hyperbolic case as well.

Recall that the covariance function of a monochromatic invariant Gaussian random field with correlation wavelength *Λ* is provided by the inverse Fourier transform of the Dirac distribution on a circle, that is,

$$\mathbb{E} \bigl[\mathbf{z}(x) \mathbf{z}(y)^{\star}\bigr] = \varGamma(x -y) = \int_{\mathbb {S}^{1}} e^{i R u \cdot(x -y)}\, du $$

with $R = \frac{2\pi}{\varLambda}$. Now, the condition on the support of *Γ* means that it satisfies the Helmholtz equation $\Delta\varGamma = -R^{2} \varGamma$; an easy consequence is that

$$\mathbb{E} \bigl[ \bigl| \Delta\mathbf{z} + R^{2} \mathbf{z} \bigr|^{2} \bigr] $$

is identically zero. This means that any (strictly speaking, almost any) orientation map drawn from **z** satisfies itself the Helmholz equation; thus maps drawn from **z** are superpositions of plane waves with wavenumber *R* and various propagation directions.

As we mentioned at the end of Sect. 2.4.3, each map drawn from **z** thus reads $f = \int_{\mathbb {S}^{1}} e^{i R u \cdot x } T_{f}(u)$, where $T_{f}$ is a complex-valued measure on $\mathbb{S}^{1}$ (the *inverse Poisson transform* of *f*). As a consequence, we know that there is a random, complex-valued Gaussian measure $d\mathbb {Z}$ on the circle which allows for describing **z** as a stochastic integral:

$$\mathbf{z}(x) = \int_{\mathbb {S}^{1}} e^{i R u \cdot x } \,d\mathbb{Z}(u). $$

Now, thanks to the Gaussian nature of **z** and the Euclidean invariance condition, there is a simple way to describe $\mathbb{Z}$, which we used for numerical simulations: the rotation invariance of **z** means that $\mathbb{Z}$ should be rotation-invariant, so it defines a stationary Gaussian process on the circle and is thus related to the Brownian bridge [36]; it has been identified by Yaglom as its mean-square derivative ([63], Eq. (3.27) to (3.30)), usually called white noise (so it is a generalized process, whose sample maps are distributions rather than continuous functions on the circle; see [72], Sect. 11.6): the Fourier expansion of $\mathbb{Z}$ is but

$$\mathbb{Z} = \theta\mapsto\sum_{n \in \mathbb {Z}} \xi_{n} e^{i n \theta}. $$

Here is a way to simulate $\mathbb{Z}$. If $(\zeta_{k})_{k \in \mathbb {N}^{\star}}$ is a sequence of independent standard Gaussian complex random variables, and if $u_{1}, \ldots, u_{n}$ are the complex numbers coding for the vertices of a regular *n*-gon inscribed in the unit circle, then

$$\mathbf{z}_{n}: x \mapsto\frac{1}{n} \sum _{i=1}^{n} \zeta_{i} e^{i R u_{i} \cdot x } $$

is a Gaussian random field. As *n* grows to infinity, we get random fields which are closer and closer to being a monochromatic invariant Gaussian random field, and our field **z** is but the limiting field.

## Appendix C: Some Results on Gaussian Random Fields on Homogeneous Spaces

### C.1 *Generalization of Theorems**A**–**D**to Nonmonochromatic Fields*

In the proof of Theorem B, the monochromaticity condition, which in the statement seems to be crucial, comes in at the last moment—it provides a simple expression for the quantity called $\lambda_{2}$ above. But now that we have the proof in hand it is easy to see how it can be adapted to evaluate the column spacing and pinwheel densities in nonmonochromatic fields also. Suppose now that **z** is a complex-valued, centered Gaussian random field on $\mathbb{D}$ whose probability distribution is $\mathit{SU}(1,1)$-invariant. Select a geodesic *δ* on $\mathbb{D}$, consider a segment *Σ* on *δ*, write $|\varSigma|_{h}$ for its hyperbolic length, *Ψ* for the real-valued random field on *Σ* obtained by projecting the values of $\mathbf{z}|_{\varSigma}$ onto an arbitrary axis in $\mathbb {C}$, and $\mathcal {N}_{\varSigma}$ for the random variable recording the number of zeroes of *Ψ* on *Σ*. Motivated by the discussion around Theorem C (see also [34]), let us define the typical spacing *Λ* as the inverse of the positive number $\frac {\mathbb{E}[ \mathcal{N}_{\varSigma} ]}{|\varSigma|_{h}}$. Because **z** is $\mathit{SU}(1,1)$-invariant, it is then independent of *δ*, *Σ*, and the choice of *Ψ*.

Recall from Sect. 3.1.3 that the covariance function of **z** can be seen as a function $\varGamma: \mathbb{D} \rightarrow \mathbb {C}$ which is rotation-invariant, and for which we can assume $\varGamma(\mathbf{1}) = 1$. Using the notations of Sect. 3.1, recall from the theorem by Harish-Chandra mentioned at the end of Sect. 3.1.2 that we can write *Γ* as $\int_{R^{+}} \varGamma_{\omega} \varPi(\lambda)\, d\omega$, where $\varGamma_{\lambda} = \int_{B} ( \int_{\mathbb{D}} \varGamma(y) e_{-\omega, b}(y)\,dy ) e_{\omega,b}(z) \,db$ is an element of $\mathcal{E}_{\omega}$. Because *Γ* is rotation-invariant, $\varGamma _{\omega}$ turns out to be rotation-invariant too, and as a consequence it is proportional to Harish-Chandra’s spherical function $\varphi_{\omega}$. But the constant depends on *Γ*, so gathering the constants and the Plancherel measure *Π* into a single measure we can write

$$\varGamma= \int_{\mathbb {R}^{+}} \varphi_{\omega} \,dP(\omega), $$

where *P* is a measure on $\mathbb {R}^{+}$. This is the spectral decomposition of the field *Γ*. Now, the following statement is what the proof of Theorem B reveals in the nonmonochromatic case.

#### Theorem B′

*Suppose***z***is a complex*-*valued*, *centered Gaussian random field on*$\mathbb{D}$*whose probability distribution is*$\mathit{SU}(1,1)$-*invariant*. *Select a geodesic**δ**on*$\mathbb{D}$, *consider a segment**Σ**on**δ*, *write*$|\varSigma|_{h}$*for its hyperbolic length*, *Ψ**for the real*-*valued random field on**Σ**obtained by projecting the values of*$\mathbf{z}|_{\varSigma}$*onto an arbitrary axis in*$\mathbb {C}$, *and*$\mathcal{N}_{\varSigma}$*for the random variable recording the number of zeroes of**Ψ**on**Σ*. *Define the typical spacing**Λ**as the inverse of the positive number*$\frac{\mathbb{E}[ \mathcal{N}_{\varSigma} ]}{|\varSigma|_{h}}$. *It is then independent of**δ*, *Σ*, *and the choice of**Ψ*. *In fact*, *in terms of the power spectrum measure**P**above*,

$$\varLambda= {2\pi} \biggl( \int_{\mathbb {R}^{+}} \bigl( \omega^{2}+1\bigr)\, dP(\lambda) \biggr)^{-1/2}. $$

Since the evaluation of the quantity called $\lambda_{2}$ in Sect. 3.1.4 was a common ingredient of both Theorems A and B, we also obtain the following analogue of Theorem A for nonmonochromatic fields.

#### Theorem A′

*Suppose***z***is a complex*-*valued*, *centered Gaussian random field on*$\mathbb{D}$*whose probability distribution is*$\mathit{SU}(1,1)$-*invariant*. *Define the typical spacing**Λ**through Theorem *B′. *Consider a Borel subset*$\mathcal {A}$*of*$\mathbb {D}$, *write*$|\mathcal {A}|_{h}$*for its area w*.*r*.*t*. *the Poincaré metric*, *and*$\mathcal {N}_{\mathcal {A}}$*for the random variable recording the number of zeroes of***z***in*$\mathcal {A}$. *Then*

$${\frac{\mathbb{E} \{ \mathcal {N}_{\mathcal {A}} \} }{|\mathcal {A}|_{h}} = \frac{\pi}{\varLambda^{2}}}. $$

Theorem A′ is the full justification for our claim that for maps sampled from invariant Gaussian fields on the hyperbolic plane, just as in the Euclidean case (see for instance [34], Result 2), a pinwheel density of *π* is a signature of symmetry.

In the spherical case, the same arguments provide analogues of Theorems C–D in the nonmonochromatic case. To give precise statements, suppose **z** is a complex-valued, centered Gaussian random field on $\mathbb{S}^{2}$ whose probability distribution is $\mathit{SO}(3)$-invariant. By taking the scalar product of **z** with the complex conjugates of spherical harmonics we obtain for each *ℓ* a Gaussian field

$$\mathbf{z}_{\ell}:= \sum_{m = -\ell}^{\ell} \biggl( \int_{\mathbb{S}^{2}} \mathbf{z}(x) \bar{Y}_{\ell,m}(x) \,dx \biggr) Y_{\ell, m} $$

on the sphere. Because of the Schur–Weyl orthogonality relations, each $\mathbf{z}_{\ell}$ is rotation-invariant and

$$\mathbf{z} = \sum_{\ell\geq0} \mathbf{z}_{\ell}. $$

But $\mathbf{z}_{\ell}$ is proportional to the field we called $\varPhi_{\ell}$ in Sect. 3.2.3, so there are real numbers $p_{\ell}$ such that

$$\mathbf{z} = \sum_{\ell\geq0} \varPhi_{\ell} p_{\ell}. $$

The power spectrum of the map in this case is the sequence $(p_{\ell })_{\ell}$, and here is the evaluation of the typical spacing.

#### Theorem C′

*Suppose***z***is a complex*-*valued*, *centered Gaussian random field on*$\mathbb{S}^{2}$*whose probability distribution is rotation*-*invariant*. *Select a geodesic**δ**on*$\mathbb{S}^{2}$, *consider a segment**Σ**on**δ*, *write*$|\varSigma |_{s}$*for its length*, *Ψ**for the real*-*valued random field on**Σ**obtained by projecting the values of*$\mathbf{z}|_{\varSigma}$*onto an arbitrary axis in*$\mathbb {C}$, *and*$\mathcal{N}_{\varSigma}$*for the random variable recording the number of zeroes of**Ψ**on**Σ*. *Define the typical spacing**Λ**as the positive number*$\frac{\mathbb{E}[ \mathcal{N}_{\varSigma} ]}{|\varSigma|_{s}}$. *It is then independent of**δ*, *Σ*, *and the choice of**Ψ*. *In fact*, *in terms of the power spectrum sequence* ($p_{\ell}$),

$$\varLambda= {2\pi} \biggl( \sum_{\ell\geq0} \ell(\ell+1) p_{\lambda} \biggr)^{-1/2}. $$

Here is the theorem that identifies a pinwheel density of *π* as a signature of symmetry.

#### Theorem D′

*Suppose***z***is a complex*-*valued*, *centered Gaussian random field on*$\mathbb{S}^{2}$*whose probability distribution is rotation*-*invariant*. *Define the typical spacing**Λ**through Theorem *C′. *Consider a Borel subset*$\mathcal {A}$*of*$\mathbb {S}^{2}$, *write*$|\mathcal {A}|_{s}$*for its area w*.*r*.*t*. *the round metric*, *and*$\mathcal {N}_{\mathcal {A}}$*for the random variable recording the number of zeroes of***z***in*$\mathcal {A}$. *Then*

$$\frac{\mathbb{E} \{ \mathcal {N}_{\mathcal {A}} \} }{|\mathcal {A}|_{s}} = \frac{\pi}{\varLambda^{2}}. $$

### C.2 *Invariant Fields on Symmetric Spaces of Higher Dimension*

In this subsection, we give the statements of some theorems which make the role of symmetry in the existence of a well-defined pinwheel density even clearer by showing what Theorems A–D become when they are generalized to higher-dimensional symmetric spaces.

Of course this has mainly a mathematical interest; higher-dimensional cortical maps are not absurd to think of (even if the cortex is a three-dimensional object, a special kind of connectivity could induce higher-dimensional maps which would exist only at a functional level; it is rather tempting to imagine that this could be the case for other senses than vision), but we only wish to clarify our mathematical conclusions here.

Recall that the symmetric spaces can be of the compact type, of the noncompact type (with negative curvature) or of flat type [52]. In the compact and noncompact type, the group acting on these spaces is semisimple; in the flat type, it is the semidirect product of a compact group with a vector group.

#### Theorem E

*Suppose***z***is a**G*-*invariant*, *monochromatic random field on a symmetric space*$G/K$*with values in*$\mathbb {R}^{\operatorname{dim}(G/K)}$. *Assume*$G/K$*is equipped with the natural Riemannian metric built from the Killing form of**G*. *Select a geodesic**δ**on*$G/K$, *consider a segment**Σ**on**δ*, *and write*$|\varSigma|$*for its length*. *Write**Ψ**for the real*-*valued random field on**Σ**obtained by projecting the values of*$\mathbf {z}|_{\varSigma}$*onto an arbitrary axis in*$\mathbb {R}^{\operatorname{dim}(G/K)}$, *and*$\mathcal{N}_{\varSigma}$*for the random variable recording the number of zeroes of**Ψ**on Σ*.

*Now*, *let us define**Λ**as the only positive number such that*$\Delta_{G/K} \mathbf{z} = \pm (\frac{4\pi^{2}}{d \cdot\varLambda^{2}} ) \mathbf{z}$. *Then*

$$\frac{\mathbb{E}[\mathcal{N}_{\varSigma}]}{|\varSigma|} = \frac{1}{\varLambda}. $$

*The *± *sign in the previous formula is a plus sign if*$G/K$*is of the compact type*, *a minus sign in the other cases*.

The proof of this theorem is essentially the same as that which we sketched for the case of the sphere $\mathbb{S}^{2}$: *Ψ* is a Gaussian field on the geodesic *δ* which can be seen (using a one-parameter subgroup of *G*) as a stationary field on the real line; to use the classical Kac–Rice formula we need only evaluate the second spectral moment of this field, and this is where the invariant Laplacian comes in.

This theorem says that the typical spacing between iso-orientation domains is, up to a simple dimensional factor, the wavelength which corresponds to the *Φ*-induced eigenvalue of the Laplacian. We stated what this gives for the sphere in the main text, and remarked that there is but a countable set of possible spacings. In the hyperbolic case, a continuum of spacings is possible, but there is a curvature-induced shift (the wavenumber has to be shifted by a “half-sum of positive roots”, as often happens in representation theory) which makes the typical spacing of a monochromatic field different from the (hyperbolic) distance between same-phase horocycles which appears in Helgason waves.

As in Theorem A′ and Theorem B′, there is a counterpart to Theorem E for nonmonochromatic fields. It is a bit less easy to write down because the Plancherel decomposition of $\mathbb{L}^{2}(G/K)$ features more than one real parameter; the extension is, however, straightforward and will appear in the Ph.D. thesis of the author.

Here is the promised extension of Theorems B and C to higher dimensions.

#### Theorem F

*With the notations of the previous theorem*, *write*$\mathcal{N}_{\mathcal{A}}$*for the random variable recording the number of zeroes of**Φ**in a Borel region*$\mathcal {A}$*of the symmetric space**X*, $|\mathcal {A}|_{G/K}$*for its volume as determined by the chosen metric on*$X = G/K$. *Then*

$$\frac{\mathbb{E}[\mathcal{N}_{\mathcal {A}}]}{|\mathcal {A}|_{G/K}} = \frac {\pi^{\operatorname{dim}(X)/ 2}}{\varLambda^{d}}. $$

This theorem says that when scaled with respect to the natural quasiperiod of a monochromatic map, the pinwheel density in a monochromatic invariant Gaussian field is always a power of *π*, and since the result is not limited to monochromatic fields, it still appears as a signature of symmetry.

We gave a full proof of this theorem for the hyperbolic case; there we could conveniently use the fact that $\mathbb{D}$ is naturally a subset of the complex plane, and that the invariant Laplacian $\Delta_{\mathbb {D}}$ has a simple expression in the usual $(x,y)$ coordinates. The ingredients for the general case are the same as those of the spherical case, namely a version of Azais and Wschebor’s theorem that travels unimpaired to general symmetric spaces, and in the compact and noncompact cases (the flat one is easy), the group-theoretical, coordinate-independent interpretation of $\Delta_{G/K}$ with the Casimir operator of the Lie algebra of *G*.

## Footnotes

1 Note that $\mathbf{z}_{\mathrm{exp}}$ is automatically continuous.

2 There are some conditions for this, called positive-definiteness, but they are automatically satisfied by correlation functions obtained from experimental data.

3 Recall that $\widehat {\varGamma}$ is rotation-invariant.

4 Likewise, if *n* is an integer, define $T(u) = u^{n} = e^{i n \vartheta }$ for every element $u = e^{i \vartheta} \in \mathbb {S}^{1}$; the circle $\mathbb {S}^{1}$ is a group under complex multiplication and $(\mathbb {C}, T)$ provides an irreducible unitary representation of $\mathbb {S}^{1}$.

5 Two given irreducible unitary representations $(\mathcal{H}_{1}, T_{1})$ and $(\mathcal{H}_{2}, T_{2})$ are *equivalent* if there is a unitary map *U* from $\mathcal{H}_{1}$ to $\mathcal{H}_{2}$ such that $UT_{1}(g)$ and $T_{2}(g) U$ coincide for every $g \in G$. In example 1, it is very easy to check that there is no such unitary map intertwining $T_{p}$ and $T_{q}$ if $p \neq q$.

6 To be precise, we can multiply $\widehat{f}$ with the Dirac distribution on the circle of radius *K*, obtaining a tempered distribution on $\mathbb {R}^{2}$, and define $f_{K}$ as the inverse Fourier transform of this multiplication: it is automatically a smooth function.

7 Since Fourier transforms of all maps in $\mathcal {E}_{\varLambda}$ are supported on a circle, we may see a function in any of the $\mathcal{E}_{\varLambda}$ as a complex-valued function on the unit circle; but the *G*-action depends on *Λ*. In this picture, any element $g = (R, \vec{x})$ of the Euclidean group gives an operator $T_{\varLambda}(g)$ on $\mathbb{L}^{2}(\mathbb {S}^{1})$:

$$T_{\varLambda}(g) \varPhi= \vec{u} \mapsto e^{i \frac{2\pi}{\varLambda} \vec {u} \cdot\vec{x}} \varPhi \bigl(R^{-1} \vec{u}\bigr); $$

and the Poisson transform $\varPhi\in\mathbb{L}^{2}(\mathbb {S}^{1}) \mapsto\int_{\mathbb {S}^{1}} \widehat{f}(K\vec{u})e^{K\vec{u} \cdot\vec{x}}\, d\vec{u} \in \mathcal {E}_{\varLambda}$ is a continuous bijection that intertwines the representation $T_{\varLambda}$ of $\mathit{SE}(2)$ with the natural representation on $\mathcal {E}_{\varLambda}$.

8 For completeness we recall that there are unitary irreducible representations of $\mathit{SU}(1,1)$ which do not enter the Plancherel decomposition of $\mathbb{L}^{2}(G/K)$; the deep and beautiful work by Bargmann and Harish-Chandra on these representations will not appear in this paper.

9 The groups $\mathit{SU}(1,1)$ and $SL_{2}(\mathbb {R})$ are famously isomorphic; see for instance [59].

10 This is a product of matrices!

11 That the eigenvalue should be real is the technical reason why the growth factor in the modulus is needed.

12 This means each $\mathbf{z}(x)$ is a complex-valued Gaussian random variable with zero mean and variance one.

13 A degenerate zero is a point at which both **z** and *d***z** are zero.

14 For legibility we rewrote the derivative in the horizontal direction as $\partial_{1}$ in the next formula: so if *φ* is a function of two variables $x_{1}, x_{2} \in\mathbb{D}$, $\partial_{1,x_{1}}$ denotes the derivative in the horizontal direction of $x_{1} \mapsto \varphi(x_{1}, x_{2})$.

15 Because translations have zero derivative, a general element $(A, v)$ of $\mathit{SE}(2)$ then acts on complex-valued functions on $\mathbb {R}^{2}$ as $f \mapsto ( x \mapsto A f( -A^{-1} v + A^{-1} x ) )$.
